# Estimating heterogeneous treatment effects with survival outcomes via a deep survival learner

**DOI:** 10.1007/s10985-026-09734-w

**Published:** 2026-10-15

**Authors:** Yuming Sun, Jian Kang, Yi Li

**Affiliations:** 1 Department of Biostatistics, University of Michigan, Ann Arbor, MI USA; 2 Department of Mathematics, William & Mary, VA Williamsburg, USA

**Keywords:** Causal inference, Conditional average treatment effect, Double robustness, Deep neural network

## Abstract

Estimating heterogeneous treatment effects in survival settings is complicated by right censoring as well as the time-varying nature of the estimand. While the conditional average treatment effect (CATE) provides a natural target, most existing approaches focus on a single prespecified time point and do not account for temporal trajectory, leading to instability in estimation. We propose a deep survival learner (DSL) for estimating heterogeneous treatment effects with right-censored outcomes. The method is based on a doubly robust pseudo-outcome whose conditional expectation identifies time-specific CATEs under standard assumptions. This construction remains unbiased if either the outcome model or the treatment assignment model is correctly specified, when properly accounting for censoring. To estimate CATEs over a clinically relevant time spectrum, DSL employs a multi-output deep neural network with shared representations, enabling joint estimation of treatment effect trajectories. From a theoretical perspective, we derive error bounds for both pointwise and joint estimation over time. We show that joint estimation can leverage temporal structure to control estimation error without incurring much additional approximation cost under smoothness conditions, leading to improved stability relative to separate estimation. Cross-fitting is incorporated to reduce overfitting and mitigate bias arising from flexible nuisance estimation. Simulation studies demonstrate favorable finite-sample performance, particularly under nuisance model misspecification. Applied to the Boston Lung Cancer Study, DSL reveals heterogeneity in the effects of perioperative chemotherapy across patient characteristics and over time.

**Supplementary Information:**

The online version contains supplementary material available at https://doi.org/10.1007/s10985-026-09734-w.

## Introduction

A substantial literature has examined heterogeneity in treatment effects, often summarized by the conditional average treatment effect (CATE) within the counterfactual framework (Imbens and Rubin 2016). Modeling CATE as a function of pre-treatment covariates enables systematic assessment of variation across patient subgroups, a central goal in precision medicine, where large-scale studies have documented marked heterogeneity in patient characteristics, disease awareness, and clinical outcomes across populations (Dharmarajan et al. 2017). In survival settings, this task is further complicated by censoring and the time-to-event nature of the outcome. Moreover, biomedical outcomes frequently arise from complex, interdependent processes involving treatment, exposure, and patient characteristics, as illustrated in HPV-related cancers (Haddad et al. 2008) and lung cancer (Zhai et al. 2022). These features challenge the adequacy of traditional parametric and semiparametric approaches. Recently, metalearner frameworks, including the M-learner, R-learner, and X-learner, have been developed. These approaches leverage the flexibility of modern machine learning methods by decomposing CATE estimation into a sequence of regression problems that can be solved with off-the-shelf algorithms. For instance, the M-learner constructs pseudo-outcomes based on inverse probability weighting (Horvitz and Thompson 1952). This modified-outcome formulation reduces the problem of estimating CATEs to a regression on covariates (Tian et al. 2014). The X-learner (Künzel et al. 2019) fits separate outcome models in the treated and control groups and uses them to impute individual treatment effects, while the R-learner (Nie and Wager 2021) formulates the problem as a residual-on-residual regression. More broadly, doubly robust learners have been developed to improve statistical efficiency and to mitigate sensitivity to nuisance model misspecification (Kennedy 2020).

Building on developments for continuous outcomes, recent work has extended metalearner approaches to survival settings. For example, Yang et al. (2025) propose a doubly robust learner that combines inverse probability of censoring weighting with nonparametric failure-time Bayesian additive regression trees to construct pseudo-outcomes for CATE estimation. Similarly, Bo et al. (2025) develop alternative pseudo-outcome constructions and extend a range of metalearners, including the X-learner, M-learner, and R-learner, to right-censored data. Beyond the metalearner framework, tree-based and forest-based methods have also been adapted to estimate heterogeneous treatment effects with survival outcomes. Cui et al. (2023) introduce causal survival forests, which extend causal forests to accommodate censoring, while Dandl et al. (2024) develop related model-based forest approaches. In a different direction, Hu (2024) propose a random-intercept accelerated failure time model combined with Bayesian additive regression trees to estimate differences in log survival time between treatment groups. Finally, Curth et al. (2021) introduce SurvITE, a neural network approach that models treatment-specific discrete-time hazard functions to capture treatment effect heterogeneity.

Several methodological challenges remain. Many pseudo-outcome based approaches rely on complete-case information and do not fully account for censoring, which can lead to bias or loss of efficiency in CATE estimation (Xu et al. 2023). Survival outcomes are inherently time-dependent, and treatment effects may evolve over follow-up due to delayed onset, attenuation, or time-varying biological mechanisms. In many applications, the primary scientific target is therefore the trajectory of heterogeneous treatment effects over a clinically relevant time interval, rather than a contrast at a single prespecified time point. Most existing methods, however, estimate CATE at a fixed time and treat different time points as unrelated, without leveraging the underlying temporal structure. This can result in unstable estimation and reduced statistical efficiency.

To address these limitations, we propose a deep survival learner (DSL) for estimating heterogeneous treatment effects with right-censored survival outcomes. We adopt a pseudo-outcome formulation that enables the use of flexible function approximators, such as deep neural networks, to learn its conditional expectation. Our approach distinguishes from existing methods by unifying causal identification, temporal modeling, and robust estimation within a single framework. First, we introduce a doubly robust pseudo-outcome for survival settings whose conditional expectation identifies the CATE on survival probabilities at each time point. The construction, when properly accounting for right censoring, is unbiased if either the outcome model or the treatment assignment model is correctly specified. Second, we cast CATE estimation as a joint learning problem over time and develop a multi-output neural network to estimate the entire treatment effect trajectory simultaneously. By sharing representations across time points, the method exploits temporal structure in the CATE, enabling information borrowing and leading to smoother and more stable estimates than separate models fit at each time point. From a theoretical perspective, we derive error bounds for both pointwise estimation and joint estimation over a time grid. We show that, under mild smoothness conditions on the treatment effect trajectory, joint estimation can control estimation error without incurring much additional approximation cost, thereby improving stability relative to separate estimation. Third, we incorporate cross-fitting to separate nuisance estimation from target estimation, which mitigates overfitting and enhances robustness when more flexible methods are used.

The remainder of the paper is organized as follows. Section 2 introduces the data structure, notation, and target estimand, and presents the proposed DNN estimator. Section 3 establishes the error rates at both a fixed time point and over a grid of time points. Section 4 reports simulations under varying degrees of nuisance model misspecification and different choices of time grids. Section 5 applies the proposed method to the Boston Lung Cancer Study, a large prospective cohort in the country, to examine heterogeneous effects of perioperative chemotherapy among patients with early-stage non-small cell lung cancer.

## Methods

### Data structure, estimand and pseudo-outcome construction

Let *T* and $\mathcal {C}$ denote the event time and censoring time, respectively. With right censoring, we observe $U=\min (T,\mathcal {C})$ and the event indicator $\Delta =\mathbb {I}(T\le \mathcal {C})$. We observe i.i.d. data $\mathcal {D}=\{(U_i,\Delta _i,X_i,W_i): i=1,\dots ,N\},$ where $X_i\in \mathbb {R}^d$ denotes pre-treatment covariates and $W_i\in \{0,1\}$ denotes treatment assignment. Under the potential outcomes framework (Imbens and Rubin 2016), let $T_i^w$ denote the potential survival time under treatment $w\in \{0,1\}$. The observed event time satisfies $T_i = W_i T_i^1 + (1-W_i) T_i^0.$ Our target estimand is the conditional average treatment effect (CATE) on the survival probability at any time *t* in the support of *U*, defined as

1 $$\begin{aligned} \tau _0(x;t) = P(T_i^1>t \mid X_i=x) - P(T_i^0>t \mid X_i=x). \end{aligned}$$

Since the potential survival times $T_i^0$ and $T_i^1$ in (1) cannot be observed simultaneously (even in the absence of censoring), direct estimation of CATE is infeasible. We propose to construct a pseudo-outcome whose expectation conditional on $X_i$ equals the target estimand:

2 $$\begin{aligned} \begin{aligned} \varphi _i(t)&= \pi _i^1(t)-\pi _i^0(t), \,\, \textrm{with} \\ \pi _i^w(t)&= S^w(X_i;t) + \frac{\mathbb {I}(W_i=w)}{e^w(X_i)} \left\{ \frac{Y_i(t)}{G^w(X_i;t)} - S^w(X_i;t) \right\} , \qquad w\in \{0,1\}, \end{aligned} \end{aligned}$$

where $S^w(x;t)$, $G^w(x;t)$, and $e^w(x)$ denote the nuisance functions corresponding to the conditional survival function $P(T_i>t\mid X_i=x,W_i=w)$, the conditional censoring survival function $P(\mathcal {C}_i>t\mid X_i=x,W_i=w)$, and the propensity score $P(W_i=w\mid X_i=x)$, respectively. Here, for a fixed time point *t*, the at-risk indicator $Y_i(t)=\mathbb {I}(U_i>t).$ In practice, these nuisance functions are unknown and must be estimated from the data, and may be subject to model misspecification.

Despite this, the pseudo-outcome in (2) remains unbiased under partial misspecification due to its double robustness. Building on this property, we estimate the pseudo-outcome by evaluating it over a prespecified grid of time points, reflecting that the target CATE is indexed by time and typically of interest over an interval rather than at a single time point. We then model its conditional expectation given baseline covariates using a deep neural network (DNN) introduced in the next subsection and obtain a flexible estimator of the time-varying CATE function.

### Deep neural network: architecture and implementation

Let $0 \le t_{\min } < t_{\max }$, where $[t_{\min }, t_{\max }]$ is a compact subset of the support of *U*, which is specified to balance clinical relevance and data support. In our motivating lung cancer study, $t_{\min }=0$ represents the time of diagnosis, whereas $t_{\max }$ is chosen to ensure that a sufficient number of patients remain at risk at that time for stable estimation. We estimate $\tau _0(x;t)$ on a prespecified grid of *J* time points, $t_{\min }=t_1< t_2< \cdots < t_J=t_{\max }.$

To model the smoothness of the treatment effects over time, we represent the time-varying CATE using a fixed basis; common choices include B-splines or indicator basis functions (de Boor 2001; Schumaker 2007). With $\{B_k(t)\}_{k = 1}^m$ being *m(≤ J)* basis functions on $[t_{\min },t_{\max }]$, we approximate the CATE by

3 $$\begin{aligned} \tau (x;t) = \sum _{k=1}^m f_k(x) B_k(t), \qquad t \in [t_{\min },t_{\max }] \end{aligned}$$

which provides a concrete low-dimensional representation of temporal variation, facilitating our later theoretical analysis. To estimate (3), we consider a fully connected feedforward network with *L* hidden layers. Let $h^{(0)}(x)=x$ and, for $\ell =1,\ldots ,L,$

$$h^{(\ell )}(x) = \sigma \!\left( W_\ell h^{(\ell -1)}(x)+b_\ell \right) ,$$

where $\sigma (u)=\max (u,0)$ is the ReLU activation applied componentwise. The *m* spline coefficient functions in (3), i.e., $f(x) = (f_1(x), f_2(x),\dots , f_m(x))^{\top }$, are jointly estimated by the output layer

$$f(x)=W_{L+1}h^{(L)}(x)+b_{L+1}\in \mathbb {R}^m.$$

Let $\mathcal {F}_{n,m}$ denote the class of such networks subject to the following structural constraints:

- (**Depth**) $$L \le L_n$$;
- (**Width**) $$\max _{1 \le \ell \le L} p_\ell \le p_n$$;
- (**Sparsity**) the total number of nonzero parameters satisfies $\sum _{\ell =1}^{L+1} \Vert W_\ell \Vert _0 + \sum _{\ell =1}^{L+1} \Vert b_\ell \Vert _0 \le s_n;$
- (**Magnitude constraint**) all entries of $\{W_\ell , b_\ell \}$ are bounded in absolute value by $B_n$.

The corresponding DNN class of time-varying CATE functions is

$$\mathcal {T}_{n,m}= \left\{ (x,t)\mapsto \sum _{k=1}^m f_k(x)B_k(t): f=(f_1,\ldots ,f_m)^\top \in \mathcal {F}_{n,m} \right\} .$$

Taking *m=1* and $B_1(t)=1$ recovers the scalar network for estimating $\tau _0(x;t)$ at a single *t*. When *J>1*, let $\textbf{B}=[B_k(t_j)]_{J\times m}$, with *m>1*, denote the fixed spline evaluation matrix. Then the CATE vector over the time grid is represented by

$$\boldsymbol{\tau }(x) = \bigl (\tau (x;t_1),\ldots ,\tau (x;t_J)\bigr )^\top =\textbf{B f}(x).$$

Thus, the spline formulation allows information to be shared across time through a common basis. As characterized in the subsequent Theorem 6, joint estimation can reduce approximation error when the CATE trajectory is sufficiently smooth in time and admits a low-complexity representation; this advantage may not hold when such temporal structure is weak or misspecified.

### Estimation

Let $\hat{\boldsymbol{\varphi }}_i = \bigl (\hat{\varphi }_i(t_1),\ldots ,\hat{\varphi }_i(t_J)\bigr )^\top$ denote the vector of estimated pseudo-outcomes for subject *i* on a prespecified grid of *J* time points, $t_{\min }=t_1< t_2< \cdots < t_J=t_{\max }.$ To reduce overfitting and permit flexible nuisance estimation, we employ *K*-fold cross-fitting throughout. Let the observed data be $\mathcal {D}=\{(U_i,\Delta _i,X_i,W_i)\}_{i=1}^N,$ and randomly partition $\mathcal {D}$ into *K* approximately equal-sized, disjoint folds $\mathcal {D}_1,\ldots ,\mathcal {D}_K.$ For each fold *k*, let $\mathcal {D}_{-k}=\mathcal {D}{\setminus } \mathcal {D}_k$ denote the corresponding training sample, and use $\mathcal {D}_k$ as the held-out sample.

Within each training sample $\mathcal {D}_{-k}$, we estimate the nuisance functions needed for pseudo-outcome construction. Specifically, for each treatment group $w\in \{0,1\}$, we estimate $S^w(x;t),$ and $G^w(x;t)$ using only subjects in $\mathcal {D}_{-k}$ with $W_i=w$. These nuisance components may be estimated using Cox-type models, or, when greater flexibility is desired, using nonparametric survival learners such as random survival forests. Also, the propensity score $e^w(x)$ is estimated on $\mathcal {D}_{-k}$ using a classification method, such as logistic regression or a more flexible tree-based ensemble learner.

These estimators are then evaluated only on observations in the held-out fold $\mathcal {D}_k$. Thus, for each subject $i\in \mathcal {D}_k$, the pseudo-outcome vector $\hat{\boldsymbol{\varphi }}_i$ is constructed using nuisance estimates obtained from data that do not include subject *i*. Repeating this procedure over all *K* folds yields cross-fitted pseudo-outcomes for every subject in the sample.

We estimate the CATE at the evaluation times as

$$\hat{\tau }(x;t_j)=\sum _{k=1}^m \hat{f}_k(x)B_k(t_j), \qquad j=1,\ldots ,J,$$

where

4 $$\begin{aligned} \hat{f} = \arg \min _{f\in \mathcal {F}_{n,m}} \frac{1}{NJ}\sum _{i=1}^N \left\| \hat{\boldsymbol{\varphi }}_i-\textbf{B f}(X_i) \right\| _2^2. \end{aligned}$$

That is, the parameters $\{W_\ell ,b_\ell \}_{\ell =1}^{L+1}$ associated with *f* are estimated by minimizing the empirical squared loss over subjects and time points. The resulting procedure, referred to as the Deep Survival Learner (DSL), is summarized in Algorithm 1.

Regardless of the specific choice of nuisance estimators, our theoretical analysis shows that the error of the DNN estimator decomposes into nuisance estimation error as well as approximation error and statistical error arising from fitting the DNN.

**Algorithm 1:**
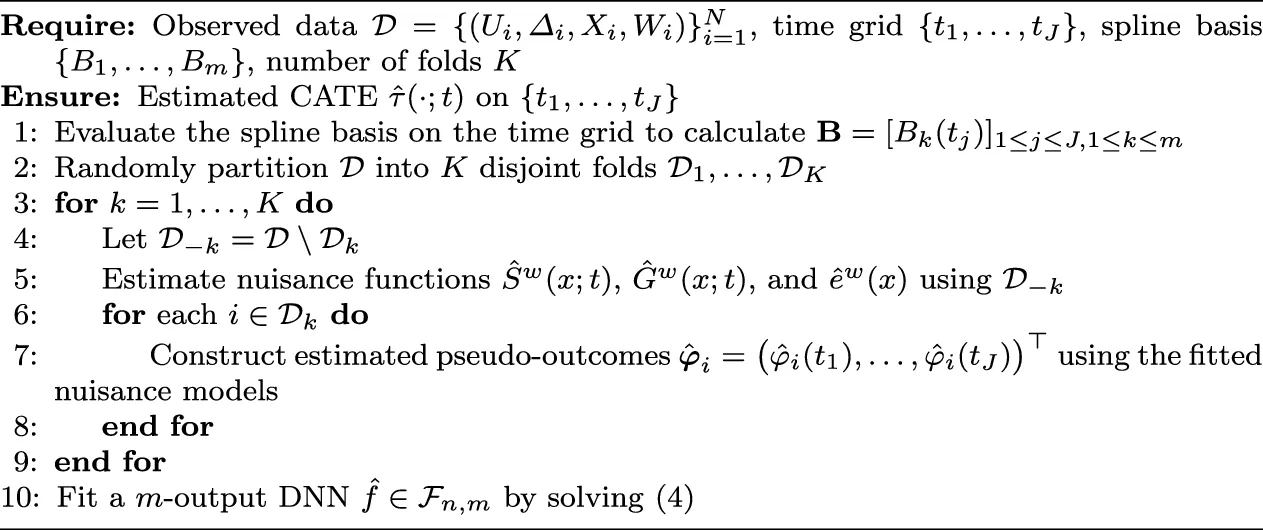
Deep survival learner (DSL)

## Theoretical properties

*Notation:* For $w \in \{0,1\}$ and $t \in [t_{\min }, t_{\max }]$, define

$$S_0^w(x;t) = P(T_i> t \mid X_i = x, W_i = w), e_0^w(x) = P(W_i = w \mid X_i = x),$$

$G_0^w(x;t) = P(\mathcal {C}_i> t \mid X_i = x, W_i = w).$ Let $\hat{S}^w(x;t)$, $\hat{e}^w(x)$, and $\hat{G}^w(x;t)$ denote the corresponding estimators. For subject *i*, let $O_i = (U_i, \Delta _i, X_i, W_i)$ denote the observed data. For any measurable function *g*, define the empirical measure by $\mathbb {P}_n g = \frac{1}{n}\sum _{i=1}^n g(O_i).$ Further, for a random function $\hat{g}(\cdot )$ constructed independently of $O_i$, the quantities $\mathbb {E}\{\hat{g}(O_i)\} \quad \text {and}\quad \mathbb {E}\{\hat{g}(O_i)\mid X_i\}$ are understood as expectations taken with respect to $O_i$ only, or $\mathbb {E}\{\hat{g}(O_i)\} = \int \hat{g}(o)\, dP_{O}(o),$ and $\mathbb {E}\{\hat{g}(O_i)|X_i=x\} = \int \hat{g}(o)\, dP_{O\mid X}(o|x).$ For a vector $x=(x_1,\ldots ,x_d)^\top \in \mathbb {R}^d$, define its $\ell _q$-norm by $\Vert x\Vert _q = \left( \sum _{j=1}^d |x_j|^q\right) ^{1/q}, \, 1\le q<\infty ,$ and $\Vert x\Vert _\infty = \max _{1\le j\le d}|x_j|.$ Let $P_X$ denote the probability distribution of the covariate vector *X* on $\mathcal {X}$. For a measurable function $f:\mathcal {X}\rightarrow \mathbb {R}$, define $\Vert f\Vert _{L_q(P_X)} = \left( \int _{\mathcal {X}} |f(x)|^q\, dP_X(x)\right) ^{1/q}, \, 1\le q<\infty ,$ and $\Vert f\Vert _{L_\infty (P_X)} = \sup _{x\in \mathcal {X}} |f(x)|.$

We impose the following assumptions to establish the statistical properties of the proposed estimator.

### Assumption 1

(*Consistency*) For each individual, $T_i = T_i^{W_i}.$

### Assumption 2

(*Unconfoundedness*) $$\{T_i^0, T_i^1\} \mathrel {\perp \!\!\!\perp }W_i \mid X_i.$$

### Assumption 3

(*Overlap*) Assume the support of *X* is $\mathcal {X}=[0,1]^d$, and $P_X$ has a density bounded away from 0 and *∞* on $\mathcal {X}$. There exists a constant *c> 0* such that $e_0^w(x) \ge c$, for all $x \in \mathcal {X}$ and $w \in \{0,1\}$.

### Assumption 4

(*Noninformative censoring*) For each $w \in \{0,1\}$, we assume that $\mathcal {C}_i \;\mathrel {\perp \!\!\!\perp }\; T_i^w \mid (X_i, W_i = w).$

### Assumption 5

(*Censoring positivity*) There exists a constant *c> 0* such that $G_0^w(x; t) \ge c$, for all (*x*, *t*) in the support of $(X_i,T_i)$ and all $w \in \{0,1\}$.

### Assumption 6

(*Uniform convergence rate*) For a fixed $t \in [t_{\min }, t_{\max }]$, assume the estimates, corresponding to $S_0^w(x;t), G_0^w(x;t)$ and $e_0^w(x)$, satisfy

$$\sup _x |\hat{S}^w(x;t)-S_0^w(x;t)|=O_p(r_n^{(S)}), \qquad \sup _x |\hat{G}^w(x;t)-G_0^w(x;t)|=O_p(r_n^{(G)}),$$

$$\sup _x |\hat{e}^w(x)-e_0^w(x)|=O_p(r_n^{(e)}),$$

for some positive sequences $r_n^{(S)}, r_n^{(G)}, r_n^{(e)}$ that converge to 0.

Assumptions 1–3 are standard in causal inference and ensure identification of the potential outcome distributions (Rosenbaum and Rubin 1983; Imbens and Rubin 2015). Assumptions 4–5 are commonly imposed in survival analysis to guarantee identifiability of the survival and censoring mechanisms (Andersen and Gill 1982; Tsiatis 2006). Assumption 6 requires uniform convergence of the estimated nuisance functions, which is standard in semiparametric and machine learning-based inference and determines the convergence rate of the proposed estimator (van der Vaart 1998; Chernozhukov et al. 2018).

### Theorem 1

(Identification) Under Assumptions 1-5, if all nuisance functions in (2) equal their true counterparts, it holds that

$$\mathbb {E}\{\varphi _i(t)\mid X_i=x\}=\tau _0(x;t).$$

Theorem 1 establishes identification of $\tau _0(x;t)$ via the conditional expectation of $\varphi _i(t)$ given $X_i$, thereby reducing the problem of estimating heterogeneous treatment effects under censoring to a conditional mean regression problem.

### Theorem 2

(Double robustness) Under Assumptions 1-5, suppose the censoring model is correctly specified, i.e., $G^w(x;t)=P(\mathcal {C}_i>t\mid X_i=x,W_i=w).$ If either the survival model is correctly specified, i.e., $S^w(x;t)=P(T_i>t\mid X_i=x,W_i=w),$ or the propensity score model is correctly specified, i.e., $e^w(x)=P(W_i=w\mid X_i=x),$ it holds that

$$\mathbb {E}\{\varphi _i(t)\mid X_i=x\}=\tau _0(x;t).$$

The double robustness of the proposed pseudo-outcome, established in Theorem 2, is especially appealing in observational survival studies, where it is often difficult to correctly specify all nuisance components.

The consistency of the final estimator further relies on the approximation and estimation properties of the DNN. We establish consistency for the scalar network estimator at a fixed time point *t* (i.e., *m=1* and $B_1(t) = 1$). We then extend the analysis to joint estimation at multiple time points with the temporal basis representation and a multi-output DNN. Recall that $\tau _0(x;t) = \mathbb {E}\big [\varphi _i^0(t)\mid X_i = x\big ],$ where $\varphi _i^0(t)$ is the oracle pseudo-outcome and $\hat{\varphi }_i(t)$ is its cross-fitted counterpart (Sect. 2.3). The DNN estimator is obtained via

$$\hat{f} = \arg \min _{f\in \mathcal {F}_{n,m}}\mathbb {P}_n\left\{ \frac{1}{J} \left\| \hat{\boldsymbol{\varphi }}_i-\textbf{B f}(X_i) \right\| _2^2\right\} .$$

where $\mathcal {F}_{n,m}$ is the DNN class. We begin with the decomposition

$$\hat{\tau }(x;t) - \tau _0(x;t) = \underbrace{\hat{\tau }(x;t) - \mathbb {E}\big [\hat{\varphi }_i(t)\mid X_i = x\big ]}_{\text {regression error}} + \underbrace{\mathbb {E}\big [\hat{\varphi }_i(t) - \varphi _i^0(t)\mid X_i = x\big ]}_{\text {nuisance-induced error}}.$$

The first term reflects the error from nonparametric regression, while the second term captures the impact of estimating nuisance functions. We bound the second term using the following results.

### Theorem 3

(Bias decomposition with estimated nuisance functions) Suppose Assumptions 3, 5, and 6 hold. Let $\hat{\varphi }_i(t)$ and $\varphi _i^0(t)$ denote the estimated and population pseudo-outcomes, respectively, obtained by plugging the cross-fitted and true nuisance functions into (2). Then, it holds that

5 $$\begin{aligned} \mathbb {E}\big [\hat{\varphi }_i(t)-\varphi _i^0(t)\mid X_i=x\big ]&= \sum _{w=0}^1 (-1)^{w+1} \Bigg [ \frac{\hat{e}^w(x)-e_0^w(x)}{\hat{e}^w(x)} \big \{\hat{S}^w(x;t)-S_0^w(x;t)\big \} \nonumber \\&+ \frac{e_0^w(x)}{\hat{e}^w(x)} \frac{S_0^w(x;t)}{\hat{G}^w(x;t)} \big \{G_0^w(x;t)-\hat{G}^w(x;t)\big \} \Bigg ]. \end{aligned}$$

Consequently, it follows that

$$\sup _x \left| \mathbb {E}\big [\hat{\varphi }_i(t)-\varphi _i^0(t)\mid X_i=x\big ] \right| = O_p\!\left( r_n^{(e)}r_n^{(S)} + r_n^{(G)}\right) .$$

In particular, if $\hat{G}^w(x;t)=G_0^w(x;t)$, then we have

$$\sup _x \left| \mathbb {E}\big [\hat{\varphi }_i(t)-\varphi _i^0(t)\mid X_i=x\big ] \right| = O_p\!\left( r_n^{(e)}r_n^{(S)}\right) .$$

We impose smoothness conditions on the CATE function in *x* and additional regularity conditions on the cross-fitted pseudo-outcomes and the network class, which are required for the consistency and error rate results at a fixed time point.

### Definition 1

(Hölder class) Let $\mathcal {X}=[0,1]^d$ and let *β>0*. Write

$$\beta =q+\alpha , \qquad q=\lfloor \beta \rfloor , \qquad \alpha \in (0,1],$$

where $q=\lfloor \beta \rfloor$ is the integer part of *β*. Let $\nu =(\nu _1,\ldots ,\nu _d)$, where each $\nu _j$ is a nonnegative integer. For $x=(x_1,\ldots ,x_d)$, define $D_x^\nu = \frac{\partial ^{\Vert \nu \Vert _1}}{\partial x_1^{\nu _1}\cdots \partial x_d^{\nu _d}}.$ For *M>0*, define $\mathcal {H}^\beta (M)$ as the class of functions $f:\mathcal {X}\rightarrow \mathbb {R}$ such that, for all mixed partial derivatives $D_x^\nu f(x)$ with $\Vert \nu \Vert _1\le q$, these derivatives exist and are uniformly bounded by *M*, that is,

$$\max _{\Vert \nu \Vert _1\le q}\sup _{x\in \mathcal {X}}|D_x^\nu f(x)|\le M,$$

and moreover,

$$\sup _{x\ne y} \frac{|D_x^\nu f(x)-D_x^\nu f(y)|}{\Vert x-y\Vert _2^\alpha } \le M, \qquad \Vert \nu \Vert _1=q.$$

### Assumption 7

(*Bounded second moments*) The cross-fitted $\hat{\varphi }_i$ satisfies $\sup _{ 1\le i \le n} \mathbb {E}\big [\hat{\varphi }_i^2(t)\big ] < \infty .$

### Assumption 8

(*Network approximation capacity*) For any fixed *m≥ 1*, smoothness parameter *β>0*, and $g_1,\ldots ,g_m\in \mathcal {H}^\beta (M)$, the network class $\mathcal {F}_{n,m}$ is rich such that there exists $f^\star = (f_1^\star ,\ldots ,f_m^\star )^\top \in \mathcal {F}_{n,m}$ satisfying

$$\max _{1\le k\le m} \Vert f_k^\star -g_k\Vert _{L_\infty (P_X)} \le C n^{-\beta /(2\beta +d)}.$$

### Assumption 9

(*Uniform convergence over network*) The empirical squared-loss process for the scalar network class $\mathcal {F}_{n,1}$ satisfies

$$\sup _{f\in \mathcal {F}_{n,1}} \left| \mathbb {P}_n\big [\{\hat{\varphi }_i(t)-f(X_i)\}^2\big ] - \mathbb {E}\big [\{\hat{\varphi }_i(t)-f(X_i)\}^2\big ] \right| = O_p(\rho _n),$$

for some $$\rho _n\rightarrow 0$$.

### Assumption 10

(*Controlled optimization error*) For fixed *J≥ 1* and *m≤ J*, let $\hat{f}=(\hat{f}_1, \ldots , \hat{f}_m)^\top \in \mathcal {F}_{n,m}$ denote the fitted *m*-output DNN. The optimization error is controlled in the sense that

$$\begin{aligned} \mathbb {P}_n\left[ \frac{1}{J}\sum _{j=1}^J \left\{ \hat{\varphi }_i(t_j)-\sum _{k=1}^m\hat{f}_k(X_i)B_k(t_j)\right\} ^2\right]&\le \inf _{f\in \mathcal {F}_{n,m}}\mathbb {P}_n\left[ \frac{1}{J}\sum _{j=1}^J \left\{ \hat{\varphi }_i(t_j)-\sum _{k=1}^m f_k(X_i)B_k(t_j)\right\} ^2\right] \\ &\qquad + \delta _{n,J,m}^2 \end{aligned}$$

where $$\delta _{n,J,m}^2 = o_p(1)$$

The Hölder class in Definition 1 is a standard smoothness condition in nonparametric regression used to characterize approximation error and minimax rates (Tsybakov 2009; Györfi et al. 2006). Assumption 7 imposes a bounded second moment condition on the pseudo-outcomes, ensuring well-behaved risk and concentration properties (Chernozhukov et al. 2018). Assumption 8 requires that the network class $\mathcal {F}_n$ can approximate $\tau _0(\cdot ;t)$ at the optimal rate, consistent with approximation results for ReLU networks under Hölder smoothness at *m=1* (Schmidt-Hieber 2020). For *m>1*, the scalar approximating networks can be combined in parallel into a single *m*-output ReLU network without changing the approximation rate in *n*. Assumption 9 ensures uniform convergence of the empirical loss to its population counterpart over $\mathcal {F}_n$, providing control of the estimation error in empirical risk minimization (van der Vaart and Wellner 1996; Bartlett et al. 2017). Finally, Assumption 10 controls the optimization error arising from approximate minimization of the empirical loss (Schmidt-Hieber 2020).

The consistency of the final estimator relies on the approximation and estimation properties of DNNs. We first consider the scalar network estimator at a fixed time *t*. This corresponds to the special case of general basis representation with *J = m =1* and $B_1(t) = 1$. We denote $\tau (x;t)= f_1(x)$, $f_1 \in \mathcal {F}_{n,1}$. We now establish consistency and convergence rate for this fixed time estimator. For the fixed-time analysis, we write $\delta _n=\delta _{n,1,1}$ for simplicity.

### Theorem 4

(Consistency of the constructed estimator at a fixed*t*) Suppose Assumptions 1– 9 hold and that the convergence rates of nuisance functions in Assumption 6 satisfy that $r_n^{(e)}r_n^{(S)}+r_n^{(G)}\rightarrow 0.$ It follows that

$$\Vert \hat{\tau }(\cdot ;t)-\tau _0(\cdot ;t)\Vert _{L_2(P_X)}\xrightarrow {p}0.$$.

### Theorem 5

(Rate for the constructed CATE estimator at a fixed *t*) Suppose $\tau _0(\cdot ;t)\in \mathcal {H}^\beta (M)$ for some *β>0* and *M>0*, and that Assumptions 1–10 hold. It follows that

$$\Vert \hat{\tau }(\cdot ;t)-\tau _0(\cdot ;t)\Vert _{L_2(P_X)} = O_p\!\left( n^{-\beta /(2\beta +d)} + \rho _n^{1/2} + \delta _n + r_n^{(e)}r_n^{(S)} + r_n^{(G)} \right) .$$

In particular, if $\rho _n\lesssim n^{-2\beta /(2\beta +d)}$, it holds that

$$\Vert \hat{\tau }(\cdot ;t)-\tau _0(\cdot ;t)\Vert _{L_2(P_X)} = O_p\!\left( n^{-\beta /(2\beta +d)} + \delta _n + r_n^{(e)}r_n^{(S)} + r_n^{(G)} \right) .$$

### Remark 1

Cross-fitting ensures that the pseudo-outcome $\hat{\varphi }_i(t)$ is constructed using nuisance estimators that are independent of observation *i*. This allows us to treat the estimated nuisance functions as fixed when taking conditional expectations, which is critical for establishing the bias decomposition in Theorem 3. In particular, it justifies the expansion $\mathbb {E}\{\hat{\varphi }_i(t)\mid X_i=x\},$ leading to the second-order term $r_n^{(e)} r_n^{(S)}$ arising from the product of estimation errors in the propensity score and outcome model. Without cross-fitting, additional first-order bias terms of order $r_n^{(e)} + r_n^{(S)}$ may appear due to overfitting. We note that estimation of the censoring model introduces an additional first-order term $r_n^{(G)}$, as reflected in the bias decomposition.

To quantify the gain from joint estimation of $\{\tau _0(x;t_j)\}_{j=1}^J$, we impose additional regularity on the CATE surface in the time dimension. In contrast to the single-time-point setting, where only smoothness in *x* is required, joint estimation exploits cross-time dependence by modeling $\tau _0(x;t)$ as a function that is jointly smooth in *(x,t)*. We formalize this through a bivariate Hölder class and prove that it induces a low-complexity representation in the time direction and enables control of the joint approximation error. The following lemma establishes this representation, and the subsequent theorem uses it to characterize the advantage of shared network estimation across time. Without loss of generality, we take $[t_{\min }, t_{\max }]=[0,1]$ throughout the development.

### Definition 2

(Hölder smoothness in *(x,t)*) Let $\mathcal {X}=[0,1]^d$ and $\mathcal {T}=[0,1]$. For $\beta _x>0$ and $\beta _t>0$, write

$$\beta _x = q_x+\alpha _x, \qquad q_x=\lfloor \beta _x\rfloor , \qquad \alpha _x\in (0,1],$$

and

$$\beta _t = q_t+\alpha _t, \qquad q_t=\lfloor \beta _t\rfloor , \qquad \alpha _t\in (0,1].$$

Let $\nu =(\nu _1,\ldots ,\nu _d)$, where $\nu _j$ is a nonnegative integer. For $x=(x_1,\ldots ,x_d)$, define $D_x^\nu = \frac{\partial ^{\Vert \nu \Vert _1}}{\partial x_1^{\nu _1}\cdots \partial x_d^{\nu _d}}.$ For *M>0*, define $\mathcal {H}^{\beta _x,\beta _t}(M)$ as the class of functions $f:\mathcal {X}\times \mathcal {T} \rightarrow \mathbb {R}$ such that, for all mixed derivatives

$$D_x^\nu \partial _t^m f(x,t), \qquad \Vert \nu \Vert _1\le q_x,\quad m=0,1,\ldots ,q_t,$$

these derivatives exist and are uniformly bounded by *M*, and moreover satisfy

$$\sup _{t\in \mathcal {T}}\sup _{x\ne y} \frac{\big |D_x^\nu \partial _t^m f(x,t)-D_x^\nu \partial _t^m f(y,t)\big |}{\Vert x-y\Vert _2^{\alpha _x}} \le M, \qquad \Vert \nu \Vert _1=q_x,\quad m=0,1,\ldots ,q_t,$$

and

$$\sup _{x\in \mathcal {X}}\sup _{s\ne t} \frac{\big |D_x^\nu \partial _t^{q_t} f(x,t)-D_x^\nu \partial _t^{q_t} f(x,s)\big |}{|t-s|^{\alpha _t}} \le M, \qquad \Vert \nu \Vert _1\le q_x.$$

### Lemma 1

(Basis expansion under Hölder smoothness) Suppose $\tau _0(\cdot ; \cdot ) \in \mathcal {H}^{\beta _x,\beta _t}(M)$ in the sense of Definition 2. Let $q_t=\lfloor \beta _t\rfloor$. For any integer *K≥ 1*, there exist $m=(q_t+1)K$ basis functions $B_1,\ldots ,B_m$ on *[0,1]* and coefficient functions $b_1,\ldots ,b_m \in \mathcal {H}^{\beta _x}(C_M):\mathcal {X}\rightarrow \mathbb {R}$ such that

$$\sup _{1\le j\le J} \left\| \tau _0(\cdot ;t_j)-\sum _{k=1}^{m} b_k(\cdot )B_k(t_j) \right\| _{L_2(P_X)}^2 \le C m^{-2\beta _t},$$

where *C>0* and $C_M>0$ depend only on the Hölder smoothness parameters. Consequently, it holds that

$$\frac{1}{J}\sum _{j=1}^J \left\| \tau _0(\cdot ;t_j)-\sum _{k=1}^{m} b_k(\cdot )B_k(t_j) \right\| _{L_2(P_X)}^2 \le C m^{-2\beta _t}.$$

### Theorem 6

(Error bound for joint estimation over time) For fixed *J>1*, let $B_1,\ldots ,B_m$, with *m≤ J*, be fixed basis functions satisfying the approximation property in Lemma 1, and let $\textbf{B}=\{B_k(t_j)\}_{1\le j\le J,\,1\le k\le m}$ denote the corresponding basis evaluation matrix.The joint estimator $\hat{\tau }(x;t) = \sum _{k=1}^m\hat{f}_k(x)B_k(t)$ is defined by

$$\hat{f} = \arg \min _{f\in \mathcal {F}_{n,m}}\mathbb {P}_n\left\{ \frac{1}{J} \left\| \hat{\boldsymbol{\varphi }}_i-\textbf{B f}(X_i) \right\| _2^2\right\}$$

where $\mathcal {F}_{n,m}$ is the class of *m*-output neural networks with shared hidden layers. Suppose $\tau _0\in \mathcal {H}^{\beta _x,\beta _t}(M)$ and that the basis stability condition holds:

$$\lambda _{\max }\left( \frac{m}{J}\textbf{B}^\top \textbf{B}\right) \le C_B.$$

Under Assumptions 1–10, then

$$\begin{aligned}&\frac{1}{J} \sum _{j=1}^J \left\| \hat{\tau }(\cdot ;t_j) - \tau _0(\cdot ;t_j) \right\| _{L_2(P_X)}^2 \\&\qquad = O_p\left( m^{-2\beta _t} + n^{-2\beta _x/(2\beta _x+d)} + \sqrt{m}\,\rho _n + \delta _{n,J,m}^2 + \left\{ r_n^{(e)}r_n^{(S)} + r_n^{(G)} \right\} ^2 \right) . \end{aligned}$$

### Remark 2

If one instead fits *J* separate DNNs, one for each time point $t_j$ without using smoothness in *t*, then the empirical process fluctuation is of order $\sqrt{J}\rho _n$. By contrast, the joint estimator exploits the temporal smoothness through a lower-dimensional representation with *m < J* basis functions and achieves a smaller empirical process fluctuation of order $\sqrt{m}\rho _n$ at the cost of temporal approximation error of order $m^{-2\beta _t}$. Therefore, the joint estimator provides a complexity advantage over fitting *J* time-specific DNNs separately when the CATE varies smoothly over time and can be well represented using *m* basis functions. This advantage need not hold when such temporal structure is weak or misspecified.

## Simulation study

To evaluate the finite-sample performance of DSL and to empirically verify its double robustness property, we conducted simulation studies under settings where either the survival model or the propensity score model is misspecified. We compared DSL with several commonly used causal machine learning methods, including the M-learner (Bo et al. 2025; Tian et al. 2014), X-learner (Künzel et al. 2019), and R-learner (Nie and Wager 2021). We also included two doubly robust learners based on nonparametric failure time Bayesian additive regression trees (NFT-BART), namely DRL-NFT-BART and PSDRL-NFT-BART (Yang et al. 2025), and the causal survival forest (Cui et al. 2023).

Across all scenarios, we generated independent datasets consisting of 800 training samples and 400 testing samples. For each subject *i*, a 30-dimensional covariate vector $X_i$ was generated independently from a uniform distribution on $[-3,3]^{30}$.

*Case 1: Correct survival and propensity score models* In this setting, both the survival model and the propensity score model were correctly specified. The treatment assignment $W_i$ was generated from a logistic regression model,

$$\operatorname {logit}\{P(W_i = 1 \mid X_i )\} = \alpha ^{\top }X_i,$$

where $\operatorname {logit}(p) = \log \{p/(1-p)\}$. The survival time followed a Weibull-Cox proportional hazards model,

$$\lambda _i(t) = \lambda \nu t^{\nu -1} \exp \big (\beta ^{\top }X_i + W_i\,\theta ^{\top }X_i\big ),$$

and the censoring time followed an Exponential-Cox model,

$$\lambda _i^C(t) = \lambda ^C \exp (\gamma ^{\top }X_i).$$

We set *λ = 0.1*, *ν = 1.2*, and $\lambda ^C = 0.2$, so the average censoring rate is approximately 35%. The coefficient vectors $\alpha , \beta , \theta , \gamma \in \mathbb {R}^{30}$ were generated as evenly spaced values on the intervals *[-1,1]*, *[0.4,-0.2]*, *[0.3,-0.1]*, and *[0.3,-0.3]*, respectively. Additional simulation results under a simplified setting with lower-dimensional covariates and a larger sample size are provided in the appendix for comparison.

*Case 2: Correct survival model, misspecified propensity score* This setting evaluates robustness to misspecification of the propensity score model. The survival and censoring mechanisms were identical to those in Case 1, while the treatment assignment followed a nonlinear model,

$$\operatorname {logit}\{P(W_i = 1 \mid X_i)\} = 0.5 + 0.45\tanh (\eta _i/1.2) + 0.25\mathbb {I}(\eta _i> 0) - 0.15\mathbb {I}(\eta _i < 1) + 0.1\sin (\eta _i^2/3),$$

where $\eta _i = \alpha ^{\top }X_i$ and *α* is defined as in Case 1. The resulting censoring rate was approximately 40%.

*Case 3: Misspecified survival model, correct propensity score* This setting evaluates robustness to misspecification of the survival model. The propensity score was generated as in Case 1, while the survival time followed a non-proportional hazards model with time-varying treatment effects,

$$\lambda _i(t) = \lambda _0 \exp \left\{ g(X_i) + W_i \cdot h(X_i)\cdot \sin \!\big (v(X_i)\, t\big ) \right\} ,$$

where

$$g(X_i) = \beta ^{\top }X_i,\quad u(X_i) = \gamma ^{\top }X_i,\quad v(X_i) = \theta ^{\top }X_i,$$

$$h(X_i) = 0.5\cos \big (u(X_i)\big ) + 0.2u(X_i)^2.$$

The coefficient vectors $\beta , \gamma , \theta$ were generated as evenly spaced values on the intervals *[-0.3,0.5]*, *[-0.2,0.3]*, and *[-0.2,0.4]*, respectively. We set $\lambda _0 = 0.05$. The censoring mechanism was the same as in Case 1, except that $\lambda ^C = 0.05$, yielding a censoring rate of approximately 50%.

In each simulation setting, we evaluated CATE over a grid of *J* time points, with $J \in \{1, 50, 100\}.$ The time grid consisted of *J* equally spaced points between the 20th and 80th percentiles of the observed survival time distribution. When *J=1*, the evaluation time corresponded to the median observed survival time.

We generated 200 independent replicates. For each replicate, DSL was trained on the training sample and evaluated on the testing sample. For replicate *r=1, … , 200*, we computed the bias and mean squared error (MSE) averaged over test subjects and time points,

$$\textrm{Bias}^{(r)} = \frac{1}{NJ}\sum _{i=1}^n\sum _{j=1}^J \Big \{ \hat{\tau }^{(r)}(X_i^{(r)};t_j^{(r)}) - \tau _0(X_i^{(r)};t_j^{(r)}) \Big \},$$

$$\textrm{MSE}^{(r)} = \frac{1}{NJ}\sum _{i=1}^n\sum _{j=1}^J \Big \{ \hat{\tau }^{(r)}(X_i^{(r)};t_j^{(r)}) - \tau _0(X_i^{(r)};t_j^{(r)}) \Big \}^2,$$

where *N = 400* denotes the number of test samples. We report the average bias and MSE across replicates, along with their corresponding 95% Monte Carlo confidence intervals, in Tables 1 and 2.

We employed five-fold cross-fitting to construct the estimated pseudo-outcomes. The nuisance functions for the survival and censoring time was estimated using Cox proportional hazards models with linear predictors, and the treatment assignment was modeled using a logistic regression with linear covariates. The DSL architecture uses the basis-expansion representation described in Section 2.2. Specifically, we use the indicator basis ($B_\ell (t_j)=I(\ell =j), \ell ,j=1,\ldots ,J$, so that the basis evaluation matrix is ($\textbf{B}=\textbf{I}_J$). Under this representation, the coefficient functions $f_1(x),\ldots ,f_J(x)$ are jointly estimated by a *J*-output DNN with shared hidden layers, resulting in $\tau (x;t_j)=f_j(x)$ at each evaluation time ($t_j$). The DNN consists of three fully connected hidden layers with 128, 64, and 32 neurons, respectively, each using ReLU activation functions, and is trained using the Adam optimizer with a learning rate of 0.003. Regularization is achieved through the architectural design of the network, including its depth and width, which implicitly control model complexity. All neural network models are implemented in PyTorch. For competing methods, the M-Learner used logistic regression (via glm) for propensity score estimation and generalized boosted regression models (GBM; gbm package) for outcome regression, with inverse probability of censoring weights estimated using random survival forests. The X-Learner employed survival random forests implemented in randomForestSRC, with tuning over terminal node sizes from 1 to 100 and candidate splitting variables starting at 15. The outcome regressions for the X-Learner were also fitted using GBM. The Causal Survival Forest was implemented using the grf package with 2000 trees and a minimum node size of 5. Finally, we implemented two doubly robust learners, DRL-NFT-BART and SDRL-NFT-BART(Yang et al. 2025) via nftbart. DRL-NFT-BART combines inverse probability of censoring weighting with outcome regression, while PSDRL-NFT-BART uses a propensity-score-weighted pseudo-outcome. Propensity scores were estimated via Super Learner, censoring probabilities via random survival forests, and NFT-BART was fitted with 1000 burn-in and 2000 posterior samples; CATE was estimated using a regression forest with 2000 trees.

Table 1 summarizes the mean bias and corresponding 95% Monte Carlo confidence intervals across all simulation settings. Overall, the proposed DSL consistently exhibits the smallest bias among competing approaches. When both the survival and treatment models are correctly specified, the confidence intervals for DSL include zero at *J=1* and *J=50*, indicating negligible bias; similar behavior persists when only the survival model is correctly specified, with coverage maintained across all *J*. In contrast, competing learners, including M-, X-, and R-learners, exhibit substantial and persistent bias that does not diminish with increasing *J*. Among the doubly robust methods, DRL-NFT-BART shows partial robustness, with bias substantially reduced under treatment model misspecification but remaining non-negligible when the survival model is misspecified. PSDRL-NFT-BART, which relies on propensity-score-weighted pseudo-outcomes, exhibits consistently large bias across all scenarios, indicating limited robustness in finite samples. When only the treatment model is correctly specified, DSL continues to outperform all competitors, achieving markedly smaller bias. For example, at *J=100*, DSL attains a bias of 0.011 (95% CI: 0.005, 0.016), compared with 0.219 (95% CI: 0.203, 0.234) for the R-learner and 0.236 (95% CI: 0.217, 0.255) for DRL-NFT-BART.

Table 2 reports the corresponding mean squared error (MSE). Across all scenarios, DSL achieves competitive performance, with only a mild increase in MSE as the number of time points *J* grows. In the fully correctly specified setting, DRL-NFT-BART and the X-learner attain slightly lower MSE than DSL, although the differences are modest. Under nuisance misspecification, DSL demonstrates a clear advantage. When only the survival model is correctly specified, DSL substantially outperforms all competing methods, with MSE increasing only slightly from 0.070 (95% CI: 0.068, 0.072) at *J=1* to 0.076 (95% CI: 0.074, 0.077) at *J=100*, compared to higher MSEs for alternatives such as the X-learner. When the survival model is misspecified, DRL-NFT-BART achieves lower MSE at small *J*, but this advantage diminishes as *J* increases, and DSL remains competitive. In contrast, PSDRL-NFT-BART exhibits consistently higher MSE across all settings, consistent with its large bias.

In summary, DSL remains competitive under correct specification, incurring minimal efficiency loss, while offering clear advantages under nuisance misspecification through reduced bias and enhanced robustness, particularly as model complexity increases.

**Table 1:** Bias of conditional average treatment effect estimators across time under nuisance model misspecification

		Case 1: correct survival Correct treatment	Case 2: correct survival Wrong treatment	Case 3: wrong survival Correct treatment
*J = 1*	Deep Survival Learner	0.002 (−0.003, 0.007)	0.003 (−0.003, 0.010)	0.012 (0.005, 0.018)
	M-Learner	0.199 (0.193, 0.204)	0.221 (0.213, 0.229)	0.305 (0.300, 0.310)
	R-Learner	0.068 (0.054, 0.082)	0.030 (0.011, 0.049)	0.223 (0.207, 0.239)
	X-Learner	0.204 (0.198, 0.211)	0.221 (0.210, 0.232)	0.297 (0.289, 0.305)
	DRL-NFT-BART	0.177 (0.161, 0.194)	−0.033 (−0.067, 0.001)	0.209 (0.188, 0.231)
	PSDRL-NFT-BART	0.360 (0.354, 0.367)	0.372 (0.364, 0.380)	0.386 (0.380, 0.391)
	Causal Survival Forest	0.378 (0.371, 0.384)	0.392 (0.382, 0.402)	0.475 (0.468, 0.481)
*J = 50*	Deep Survival Learner	−0.001 (−0.006, 0.004)	0.001 (−0.006, 0.007)	0.012 (0.006, 0.018)
	M-Learner	0.199 (0.194, 0.204)	0.221 (0.213, 0.229)	0.305 (0.299, 0.310)
	R-Learner	0.068 (0.054, 0.081)	0.022 (0.003, 0.041)	0.220 (0.205, 0.234)
	X-Learner	0.206 (0.200, 0.213)	0.226 (0.216, 0.236)	0.300 (0.292, 0.308)
	DRL-NFT-BART	0.167 (0.147, 0.187)	−0.024 (−0.056, 0.009)	0.238 (0.221, 0.255)
	PSDRL-NFT-BART	0.360 (0.354, 0.366)	0.373 (0.364, 0.381)	0.386 (0.380, 0.392)
	Causal Survival Forest	0.378 (0.372, 0.384)	0.393 (0.383, 0.403)	0.474 (0.468, 0.481)
*J = 100*	Deep Survival Learner	−0.007 (−0.012, −0.002)	−0.001 (−0.008, 0.005)	0.011 (0.005, 0.016)
	M-Learner	0.198 (0.193, 0.204)	0.221 (0.213, 0.229)	0.305 (0.299, 0.310)
	R-Learner	0.073 (0.061, 0.086)	0.028 (0.010, 0.047)	0.219 (0.203, 0.234)
	X-Learner	0.205 (0.198, 0.212)	0.229 (0.219, 0.240)	0.295 (0.286, 0.303)
	DRL-NFT-BART	0.158 (0.136, 0.180)	−0.037 (−0.071, −0.002)	0.236 (0.217, 0.255)
	PSDRL-NFT-BART	0.360 (0.354, 0.367)	0.373 (0.364, 0.381)	0.384 (0.378, 0.389)
	Causal Survival Forest	0.378 (0.372, 0.384)	0.393 (0.384, 0.403)	0.474 (0.468, 0.481)

^1^ Mean bias and 95% Monte Carlo confidence intervals are computed based on 200 simulation replicates. ^2^ Conditional average treatment effects are evaluated at $J \in \{1, 50, 100\}$ time points, equally spaced between the 20th and 80th percentiles of the observed survival time distribution. ^3^ Columns correspond to combinations of correctly and incorrectly specified survival and treatment assignment models; the censoring model is correctly specified in all scenarios

**Table 2:** Mean squared error of conditional average treatment estimators across time under nuisance model misspecification

		Case 1: Correct Survival Correct Treatment	Case 2: Correct Survival Wrong Treatment	Case 3: Wrong Survival Correct Treatment
*J = 1*	Deep Survival Learner	0.077 (0.075, 0.080)	0.070 (0.068, 0.072)	0.104 (0.101, 0.106)
	M-Learner	0.114 (0.110, 0.117)	0.122 (0.117, 0.127)	0.190 (0.185, 0.194)
	R-Learner	0.174 (0.168, 0.180)	0.235 (0.227, 0.244)	0.260 (0.251, 0.269)
	X-Learner	0.081 (0.078, 0.084)	0.093 (0.089, 0.098)	0.154 (0.150, 0.159)
	DRL-NFT-BART	0.070 (0.064, 0.075)	0.133 (0.113, 0.153)	0.087 (0.077, 0.097)
	PSDRL-NFT-BART	0.142 (0.138, 0.146)	0.152 (0.147, 0.157)	0.152 (0.148, 0.156)
	Causal Survival Forest	0.156 (0.151, 0.160)	0.170 (0.163, 0.176)	0.229 (0.222, 0.235)
*J = 50*	Deep Survival Learner	0.084 (0.082, 0.086)	0.074 (0.072, 0.076)	0.112 (0.109, 0.115)
	M-Learner	0.114 (0.110, 0.117)	0.122 (0.117, 0.127)	0.189 (0.185, 0.194)
	R-Learner	0.174 (0.168, 0.180)	0.237 (0.229, 0.245)	0.261 (0.251, 0.270)
	X-Learner	0.083 (0.080, 0.085)	0.094 (0.090, 0.098)	0.157 (0.152, 0.161)
	DRL-NFT-BART	0.077 (0.062, 0.091)	0.126 (0.108, 0.143)	0.087 (0.081, 0.094)
	PSDRL-NFT-BART	0.141 (0.137, 0.146)	0.152 (0.147, 0.158)	0.152 (0.148, 0.156)
	Causal Survival Forest	0.156 (0.152, 0.160)	0.170 (0.164, 0.177)	0.228 (0.222, 0.235)
*J = 100*	Deep Survival Learner	0.084 (0.082, 0.086)	0.076 (0.074, 0.077)	0.114 (0.111, 0.117)
	M-Learner	0.114 (0.110, 0.117)	0.123 (0.118, 0.128)	0.190 (0.185, 0.194)
	R-Learner	0.173 (0.167, 0.179)	0.233 (0.225, 0.241)	0.260 (0.252, 0.269)
	X-Learner	0.082 (0.080, 0.085)	0.096 (0.091, 0.100)	0.154 (0.150, 0.158)
	DRL-NFT-BART	0.082 (0.066, 0.098)	0.139 (0.117, 0.161)	0.088 (0.082, 0.094)
	PSDRL-NFT-BART	0.142 (0.138, 0.146)	0.152 (0.147, 0.158)	0.150 (0.146, 0.155)
	Causal Survival Forest	0.156 (0.152, 0.161)	0.171 (0.164, 0.177)	0.228 (0.222, 0.234)

^1^ Mean squared error and 95% Monte Carlo confidence intervals are computed based on 200 simulation replicates. ^2^ Conditional average treatment effects are evaluated at $J \in \{1, 50, 100\}$ time points, equally spaced between the 20th and 80th percentiles of the observed survival time distribution. ^3^ Columns correspond to combinations of correctly and incorrectly specified survival and treatment assignment models; the censoring model is correctly specified in all scenarios

## Boston lung cancer study

We apply the proposed method to investigate heterogeneous treatment effects of perioperative chemotherapy using data from the Boston Lung Cancer Study (BLCS), a large prospective longitudinal cancer cohort initiated in 1992 (Alvarez et al., 2026; Wang et al., 2023; Zhai et al., 2022). The BLCS has enrolled over 12,000 patients treated at Massachusetts General Hospital and includes detailed clinical, pathological, and follow-up information, including treatment history and survival outcomes.

We evaluate the effect of perioperative chemotherapy on overall survival among patients with non–small cell lung cancer (NSCLC). The primary outcome is the time from diagnosis to death or censoring. Because perioperative chemotherapy is primarily administered to patients with early-stage disease, we restrict the analysis to patients with stage II or III NSCLC who underwent surgical resection. Within this subset, we compare patients who received perioperative chemotherapy with those who did not. Patients with missing information on cancer histology, stage, treatment status, or diagnosis date are excluded. After applying these inclusion and exclusion criteria, a total of 784 patients from the BLCS cohort are included in the analysis.

**Fig. 1:**
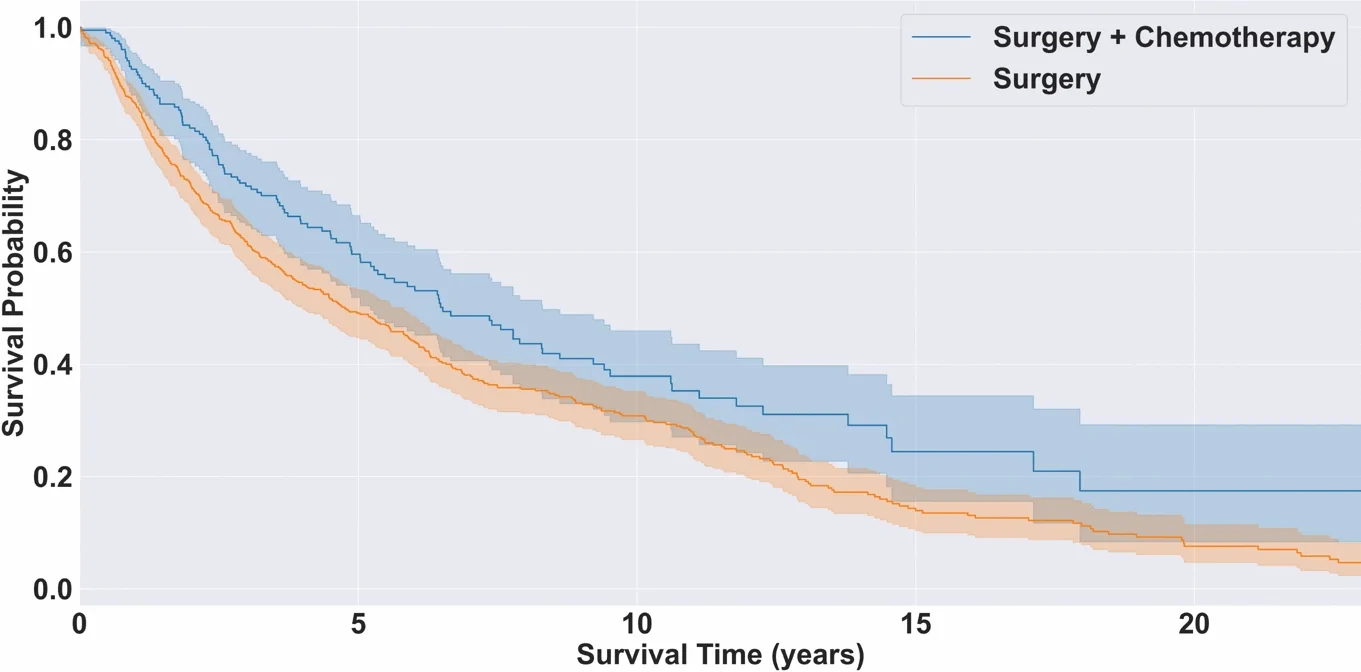
Kaplan–Meier survival curves by treatment group

Based on the descriptive statistics in Table 3, the study cohort consists of 784 patients, among whom 521 (66%) deaths were observed during follow-up. The median survival time for the overall population is 5.4 years (95% CI: 4.7-−6.1). Patients who received surgery plus perioperative chemotherapy are defined as the treatment group, whereas those who underwent surgery alone constitute the control group. A total of 214 patients (27%) received perioperative chemotherapy. Baseline characteristics differ between the two groups. Patients in the treatment group are younger (median age 64 years, IQR: 58–71) than those in the control group (median age 68 years, IQR: 60–75), and a higher proportion of treated patients are male (54% vs. 48%). Survival outcomes appear more favorable in the treatment group: 110 deaths (51%) were observed among treated patients compared with 411 deaths (72%) in the control group. The median survival time is 6.5 years (95% CI: 5.3-−8.6) for the treatment group, versus 4.8 years (95% CI: 4.1-−5.8) for the control group. Missing values in age, BMI, and smoking intensity are imputed using their respective sample means (mean imputation). Kaplan–Meier curves (Fig. 1) further illustrate differences in survival probabilities between patients who received perioperative chemotherapy and those who did not.

**Table 3:** Clinical characteristics of patients from the boston lung cancer cohort

Characteristic	Overall N = 784^1^	Surgery N = 570^1^	Surgery + Chemotherapy N = 214^1^
Death	521 (66%)	411 (72%)	110 (51%)
Median survival time (years)	5.4 (4.7, 6.1)	4.8 (4.1, 5.8)	6.5 (5.3, 8.6)
Age at diagnosis (yrs)	67 (59, 74)	68 (60, 75)	64 (58, 71)
Unknown	19	12	7
BMI (kg/m^2^)	25.8 (22.9, 29.5)	25.8 (22.8, 29.6)	26.3 (23.5, 29.5)
Unknown	60	53	7
Tumor stage			
II	445 (57%)	333 (58%)	112 (52%)
III	339 (43%)	237 (42%)	102 (48%)
Gender			
Female	394 (50%)	296 (52%)	98 (46%)
Male	390 (50%)	274 (48%)	116 (54%)
Race			
White	746 (95%)	547 (96%)	199 (93%)
Asian	15 (1.9%)	7 (1.2%)	8 (3.7%)
Black	8 (1.0%)	5 (0.9%)	3 (1.4%)
Other	15 (1.9%)	11 (1.9%)	4 (1.9%)
Smoking status			
Current smoker	213 (27%)	166 (29%)	47 (22%)
Former smoker	490 (62%)	353 (62%)	137 (64%)
Never smoker	81 (10%)	51 (8.9%)	30 (14%)

^1^Median (IQR); n (%)

We estimate CATEs at 10 prespecified time points ranging from 1 to 23 years after surgery. At each time point *t*, the CATE is defined as the difference in survival probabilities at time *t* between surgery plus perioperative chemotherapy and surgery alone, conditional on pre-treatment covariates. In our implementation, DSL employs an indicator basis over the 10 prespecified evaluation time points, such that $B_\ell (t_j)=I(\ell =j)$ and the corresponding basis evaluation matrix is $\textbf{B}=\textbf{I}_{10}$. Thus, the 10 coefficient functions are jointly estimated by a 10-output DNN with shared hidden layers. The DNN consists of three-layer feedforward network with hidden layers of sizes 64, 32, and 16, respectively, and uses ReLU activation functions.

**Fig. 2:**
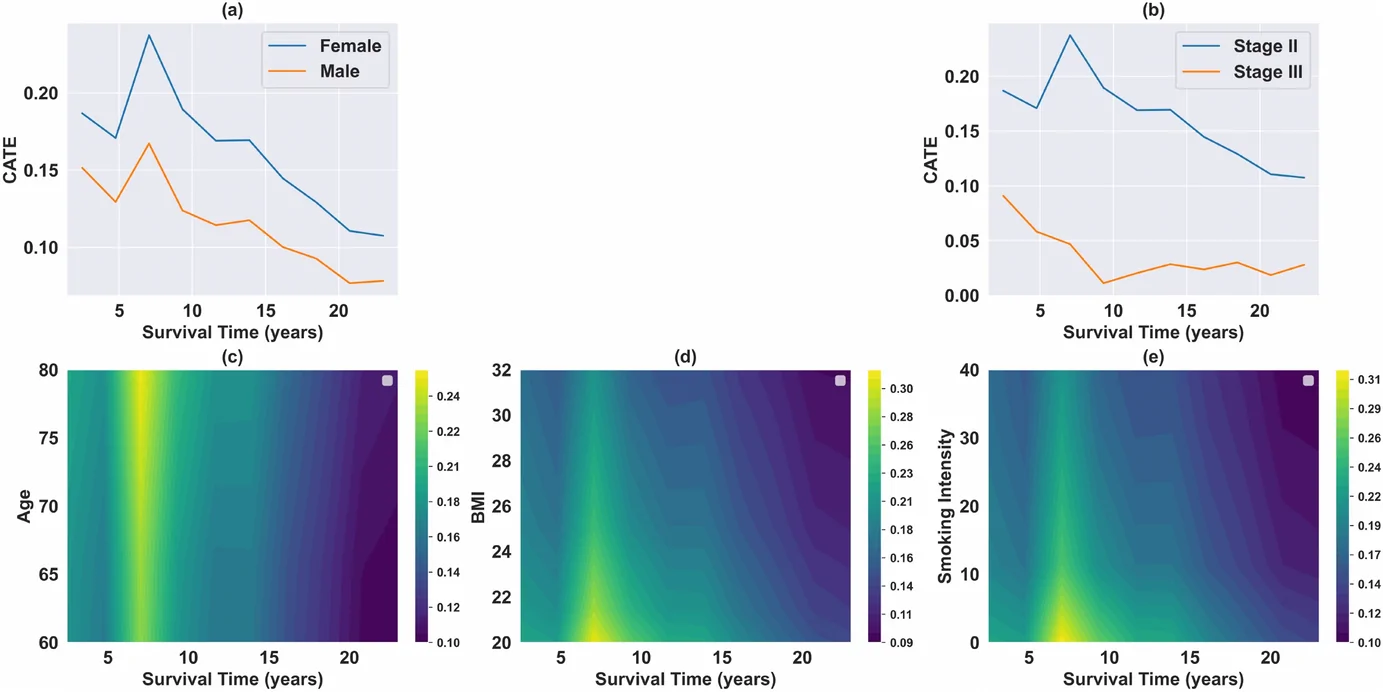
Estimated conditional average treatment effects across key baseline covariates. Each panel displays the estimated CATE as a function of one covariate of interest (gender, tumor stage, age, body mass index, or smoking intensity), with all remaining covariates fixed at their sample means or modal values

Figure 2 presents the estimated CATEs across key baseline covariates, including gender, tumor stage, age, body mass index (BMI), and smoking intensity. Across all subgroups, perioperative chemotherapy is associated with positive treatment effects over the time horizon considered, corresponding to higher survival probabilities relative to surgery alone. The magnitude of the estimated CATE increases during the first ten years following surgery and attenuates thereafter.

Gender-specific results (Fig. 2a) indicate larger treatment effects for female patients. Holding other covariates at their mean or mode, the estimated increases in 5-, 10-, and 20-year survival probabilities are 3.9%, 9.7%, and 6.8% for female patients, compared with 2.7%, *-2.8%*, and *-0.5%* for male patients. Substantial heterogeneity is also observed by tumor stage (Fig. 2b). For patients with stage II NSCLC, perioperative chemotherapy is associated with increases of 3.9%, 9.7%, and 6.8% in 5-, 10-, and 20-year survival probabilities, respectively. In contrast, the estimated effects for stage III patients are smaller, with corresponding increases of 3.3%, 3.7%, and 3.3%.

Age-specific effects (Fig. 2c) are relatively stable. Holding other covariates at their mean or mode, the estimated increases in 5-, 10-, and 20-year survival probabilities are 3.9%, 10.1%, and 7.1% for patients aged 65 years, and 3.6%, 6.7%, and 5.1% for patients aged 75 years. Figure 2d suggests that patients with higher BMI experience larger treatment effects: patients with BMI *=32* show increases of 4.0%, 10.1%, and 7.1% at 5, 10, and 20 years, compared with 3.8%, 8.1%, and 5.9% for patients with BMI *=22*. Smoking intensity also modifies treatment effects (Fig. 2e). Patients smoking 10 cigarettes per day exhibit larger survival gains (4.3%, 13.2%, and 8.9%) than those smoking 30 cigarettes per day (3.7%, 7.6%, and 5.6%). Overall, perioperative chemotherapy is associated with meaningful survival benefits for patients with early-stage NSCLC, with effects concentrated in the first ten years following surgery and attenuating thereafter. This temporal pattern is consistent with long-term follow-up from the International Adjuvant Lung Cancer Trial, which reported a significant interaction between treatment and follow-up time, with benefits diminishing beyond five years (Arriagada et al. 2010). The observed heterogeneity by gender and tumor stage aligns with prior studies reporting greater benefits among female patients (Leiter et al. 2022; Sandler et al. 2016) and stronger effects in stage II disease relative to more advanced stages (Douillard et al. 2006). The findings on smoking intensity are also consistent with prior evidence linking lower smoking exposure to improved chemotherapy outcomes (Zhang et al. 2008). Finally, patients with higher BMI appear to benefit more from perioperative chemotherapy, a pattern consistent with the reported obesity paradox in lung cancer (Sakin et al. 2021). These results underscore the importance of accounting for treatment effect heterogeneity and equity in evaluating treatment effectiveness (Arriagada et al. 2010; Leiter et al. 2022; Sandler et al. 2016; Douillard et al. 2006; Zhang et al. 2008).

## Discussion

We propose the Deep Survival Learner (DSL), a causal deep learning framework for estimating heterogeneous treatment effects with right-censored survival outcomes. The key contribution is the integration of a doubly robust pseudo-outcome for time-specific conditional average treatment effects with joint learning across time via shared representations. This formulation directly targets treatment effect trajectories over clinically relevant follow-up intervals and improves efficiency relative to pointwise approaches that ignore temporal dependence.

The method relies on standard identification conditions, including unconfoundedness and appropriate handling of censoring. While the doubly robust construction mitigates misspecification of either the outcome or treatment model, correct specification of the censoring mechanism remains essential. This highlights the need for extensions that relax noninformative censoring, for example through joint modeling, sensitivity analysis, or alternative identification strategies.

From a statistical perspective, DSL performs joint estimation of a vector-valued target over a time grid, trading bias and variance through shared representations. This raises questions on optimal grid design, regularization, and approximation error in high-dimensional settings. Extensions to competing risks would further broaden the framework to multivariate event processes and require redefining target functionals.

Finally, our current analysis focuses on point estimation. Developing valid uncertainty quantification for time-indexed CATEs under censoring remains an important open problem, requiring new theoretical tools that account for dependence across time and the complexity of deep learners.

## Supplementary Information

Below is the link to the electronic supplementary material.

Supplementary file 1 (pdf 320 KB)
