# Supplementary material for: Estimating heterogeneous treatment effects with survival outcomes via a deep survival learner
Source: Lifetime Data Anal. 2026 Oct 15;32(4):54. doi: 10.1007/s10985-026-09734-w (PMC13672394; doi:10.1007/s10985-026-09734-w)
Supplement: Supplementary file 1 [file 10985_2026_9734_MOESM1_ESM.pdf]

Supplementary Materials for
"estimating heterogeneous treatment effects with survival outcomes via a deep survival learner"

Proofs of Theorems

Proof of Theorem 1: It suffices to show that, for each $w \in \{0,1\}$,

$$\mathbb{E}\{\pi_i^w(t) \mid X_i = x\} = P(T_i^w > t \mid X_i = x),$$

since this immediately implies

$$\mathbb{E}\{\varphi_i(t) \mid X_i = x\} = P(T_i^1 > t \mid X_i = x) - P(T_i^0 > t \mid X_i = x) = \tau_0(x;t).$$

We prove the result for $w = 1$; the case $w = 0$ follows by symmetry.

Recall that

$$\pi_i^1(t) = S^1(x;t) + \frac{\mathbb{I}(W_i = 1)}{e^1(x)} \left\{ \frac{\mathbb{I}(U_i > t)}{G^1(x;t)} - S^1(x;t) \right\},$$

where $S^1(x;t) = P(T_i > t \mid X_i = x, W_i = 1)$, $e^1(x) = P(W_i = 1 \mid X_i = x)$, and $G^1(x;t) = P(\mathcal{C}_i > t \mid X_i = x, W_i = 1)$.

Taking conditional expectation given $X_i = x$ yields

$$\mathbb{E}\{\pi_i^1(t) \mid X_i = x\} = S^1(x;t) + \mathbb{E}\left[\frac{\mathbb{I}(W_i = 1)}{e^1(x)} \left\{ \frac{\mathbb{I}(U_i > t)}{G^1(x;t)} - S^1(x;t) \right\} \middle| X_i = x \right].$$

By the law of iterated expectations, the second term can be written as

$$\mathbb{E}\left[\mathbb{E}\left\{ \frac{\mathbb{I}(W_i = 1)}{e^1(x)} \left(\frac{\mathbb{I}(U_i > t)}{G^1(x;t)} - S^1(x;t) \right) \middle| X_i = x, W_i \right\} \middle| X_i = x \right].$$

Conditioning on W_i, and noting that $\mathbb{I}(W_i = 1)$ is deterministic given W_i, we obtain

$$= \mathbb{E}\left[\mathbb{I}(W_i = 1) \frac{1}{e^1(x)} \left\{ \mathbb{E}\left[\frac{\mathbb{I}(U_i > t)}{G^1(x;t)} \middle| X_i = x, W_i = 1 \right] - S^1(x;t) \right\} \middle| X_i = x \right].$$

Using the definition $U_i = \min(T_i, \mathcal{C}_i)$, we have $\mathbb{I}(U_i > t) = \mathbb{I}(T_i > t, \mathcal{C}_i > t)$. Under Assumption 4, $\mathcal{C}_i \perp T_i^w \mid (X_i, W_i = w)$ for $w \in \{0,1\}$. Under Assumption 1, when conditioning on $W_i = w$ we have $T_i = T_i^w$, which implies $\mathcal{C}_i \perp T_i \mid (X_i, W_i = w)$, and in particular, $T_i \perp \mathcal{C}_i \mid (X_i, W_i = 1)$. This implies

$$\mathbb{E}[\mathbb{I}(T_i > t, \mathcal{C}_i > t) | X_i = x, W_i = 1] = P(T_i > t \mid X_i = x, W_i = 1) P(\mathcal{C}_i > t \mid X_i = x, W_i = 1).$$

Therefore,

$$\mathbb{E}\left[\frac{\mathbb{I}(U_i > t)}{G^1(x;t)} \middle| X_i = x, W_i = 1 \right] = \frac{P(T_i > t \mid X_i = x, W_i = 1) P(\mathcal{C}_i > t \mid X_i = x, W_i = 1)}{P(\mathcal{C}_i > t \mid X_i = x, W_i = 1)} = S^1(x;t).$$

Substituting back yields

$$\mathbb{E}\{\pi_i^1(t) \mid X_i = x\} = S^1(x;t) + \mathbb{E}\left[\mathbb{I}(W_i = 1)\frac{1}{e^1(x)}\{S^1(x;t) - S^1(x;t)\}\middle| X_i = x\right] \\ = S^1(x;t).$$

Finally, by Assumptions 1 and 2,

$$S^1(x;t) = P(T_i > t \mid X_i = x, W_i = 1) = P(T_i^1 > t \mid X_i = x).$$

This completes the proof.

Proof of Theorem 2: It suffices to show that for each $w \in \{0, 1\}$,

$$\mathbb{E}\{\pi_i^w(t) \mid X_i = x\} = P(T_i^w > t \mid X_i = x),$$

because then $\mathbb{E}\{\varphi_i(t) \mid X_i = x\} = P(T_i^1 > t \mid X_i = x) - P(T_i^0 > t \mid X_i = x) = \tau_0(x;t)$. We prove the claim for $w = 1$; the case $w = 0$ follows by symmetry.

Recall the definition

$$\pi_i^1(t) = S^1(X_i;t) + \frac{\mathbb{I}(W_i = 1)}{e^1(X_i)}\left\{\frac{Y_i(t)}{G^1(X_i;t)} - S^1(X_i;t)\right\},$$

where $Y_i(t) = \mathbb{I}(U_i > t) = \mathbb{I}(T_i > t, \mathcal{C}_i > t)$, and where the nuisance functions $S^1(x;t)$, $G^1(x;t)$ and $e^1(x)$ are intended to approximate

$$S_0^1(x;t) = P(T_i > t \mid X_i = x, W_i = 1), \qquad G_0^1(x;t) = P(\mathcal{C}_i > t \mid X_i = x, W_i = 1),$$
$$e_0^1(x) = P(W_i = 1 \mid X_i = x).$$

Throughout, Assumptions 1–5 ensure that these conditional probabilities are well-defined and that $G_0^1(x;t) > 0$.

Case A: the survival model is correctly specified. Assume $S^1(x;t) = S_0^1(x;t)$ and the censoring model is correctly specified, i.e., $G^1(x;t) = G_0^1(x;t)$. Then, regardless of whether the propensity score model $e^1(\cdot)$ is correct, the argument in Theorem 1 applies verbatim and yields

$$\mathbb{E}\{\pi_i^1(t) \mid X_i = x\} = S_0^1(x;t) = P(T_i^1 > t \mid X_i = x),$$

where the last equality follows from Assumptions 1 and 2. For completeness, we provide the key step:

$$\mathbb{E}\left[\frac{Y_i(t)}{G_0^1(x;t)}\middle| X_i = x, W_i = 1\right] = \frac{P(T_i > t, \mathcal{C}_i > t \mid X_i = x, W_i = 1)}{P(\mathcal{C}_i > t \mid X_i = x, W_i = 1)} = P(T_i > t \mid X_i = x, W_i = 1),$$

where the second equality uses Assumption 4 (noninformative censoring), i.e., $T_i \perp \mathcal{C}_i \mid (X_i, W_i = 1)$. This establishes unbiasedness in Case A.

Case B: the propensity score model is correctly specified. Now assume the censoring model is correctly specified, $G^1(x;t) = G_0^1(x;t)$, and the propensity score is correctly specified, $e^1(x) = e_0^1(x) = P(W_i = 1 \mid X_i = x)$,

while allowing the survival model $S^1(x;t)$ to be misspecified. We show that $\mathbb{E}\{\pi_i^1(t) \mid X_i = x\} = S_0^1(x;t)$.

Condition on $X_i = x$. Using the definition of $\pi_i^1(t)$,

$$\mathbb{E}\{\pi_i^1(t) \mid X_i = x\} = S^1(x;t) + \mathbb{E}\left[\frac{\mathbb{I}(W_i = 1)}{e_0^1(x)} \left\{ \frac{Y_i(t)}{G_0^1(x;t)} - S^1(x;t) \right\} \middle| X_i = x \right].$$

Apply the law of iterated expectations conditioning on (X_i, W_i):

$$\begin{aligned} &\mathbb{E}\left[\frac{\mathbb{I}(W_i = 1)}{e_0^1(x)} \left\{ \frac{Y_i(t)}{G_0^1(x;t)} - S^1(x;t) \right\} \middle| X_i = x \right] \\ &\quad = \mathbb{E}\left[\mathbb{E}\left\{ \frac{\mathbb{I}(W_i = 1)}{e_0^1(x)} \left(\frac{Y_i(t)}{G_0^1(x;t)} - S^1(x;t) \right) \middle| X_i = x, W_i \right\} \middle| X_i = x \right]. \end{aligned}$$

Inside the inner expectation, $\mathbb{I}(W_i = 1)$ is fixed given W_i. Hence

$$= \mathbb{E}\left[\frac{\mathbb{I}(W_i = 1)}{e_0^1(x)} \left\{ \mathbb{E}\left[\frac{Y_i(t)}{G_0^1(x;t)} \middle| X_i = x, W_i = 1 \right] - S^1(x;t) \right\} \middle| X_i = x \right].$$

We next compute the conditional mean term. Since $Y_i(t) = \mathbb{I}(T_i > t, \mathcal{C}_i > t)$ and $G_0^1(x;t) = P(\mathcal{C}_i > t \mid X_i = x, W_i = 1)$,

$$\mathbb{E}\left[\frac{Y_i(t)}{G_0^1(x;t)} \middle| X_i = x, W_i = 1 \right] = \frac{\mathbb{E}[\mathbb{I}(T_i > t, \mathcal{C}_i > t) \mid X_i = x, W_i = 1]}{P(\mathcal{C}_i > t \mid X_i = x, W_i = 1)}.$$

By Assumption 4 and a similar argument in the Proof of Theorem 1, $T_i \perp \mathcal{C}_i \mid (X_i, W_i = 1)$, so

$$P(T_i > t, \mathcal{C}_i > t \mid X_i = x, W_i = 1) = P(T_i > t \mid X_i = x, W_i = 1)\, P(\mathcal{C}_i > t \mid X_i = x, W_i = 1).$$

Therefore,

$$\mathbb{E}\left[\frac{Y_i(t)}{G_0^1(x;t)} \middle| X_i = x, W_i = 1 \right] = P(T_i > t \mid X_i = x, W_i = 1) = S_0^1(x;t).$$

Substituting this back,

$$\begin{aligned} \mathbb{E}\{\pi_i^1(t) \mid X_i = x\} &= S^1(x;t) + \mathbb{E}\left[\frac{\mathbb{I}(W_i = 1)}{e_0^1(x)} \left\{ S_0^1(x;t) - S^1(x;t) \right\} \middle| X_i = x \right] \\ &= S^1(x;t) + \left\{ S_0^1(x;t) - S^1(x;t) \right\} \mathbb{E}\left[\frac{\mathbb{I}(W_i = 1)}{e_0^1(x)} \middle| X_i = x \right]. \end{aligned}$$

Because $e_0^1(x) = P(W_i = 1 \mid X_i = x)$, we have

$$\mathbb{E}\left[\frac{\mathbb{I}(W_i = 1)}{e_0^1(x)} \middle| X_i = x \right] = \frac{\mathbb{E}\{\mathbb{I}(W_i = 1) \mid X_i = x\}}{e_0^1(x)} = \frac{P(W_i = 1 \mid X_i = x)}{P(W_i = 1 \mid X_i = x)} = 1.$$

This identity follows from correct specification of the propensity score and Assumption 3, which guarantees $0 < e_0^1(x) < 1$. Hence,

$$\mathbb{E}\{\pi_i^1(t) \mid X_i = x\} = S_0^1(x;t) = P(T_i > t \mid X_i = x, W_i = 1).$$

Finally, by Assumptions 1 and 2,

$$P(T_i > t \mid X_i = x, W_i = 1) = P(T_i^1 > t \mid X_i = x),$$

so $\mathbb{E}\{\pi_i^1(t) \mid X_i = x\} = P(T_i^1 > t \mid X_i = x)$, completing the proof for $w = 1$.

The proof for $w = 0$ is identical after replacing 1 by 0 throughout.

Proof of Theorem 3: We first analyze the treatment-specific component $\hat{\pi}_i^w(t)$ for fixed $w \in \{0, 1\}$. As defined in the notation section, conditioning on $X_i = x$, and using cross-fitting so that $\hat{S}^w(x;t)$, $\hat{e}^w(x)$, and $\hat{G}^w(x;t)$ are fixed, we take expectation with respect to $T_i, \mathcal{C}_i$:

$$\mathbb{E}\{\hat{\pi}_i^w(t) \mid X_i = x\} = \hat{S}^w(x;t) + \frac{1}{\hat{e}^w(x)} \mathbb{E}\left[\mathbb{I}(W_i = w) \left\{ \frac{Y_i(t)}{\hat{G}^w(x;t)} - \hat{S}^w(x;t) \right\} \middle| X_i = x \right].$$

By iterated expectation,

$$\begin{aligned} &\mathbb{E}\left[\mathbb{I}(W_i = w) \left\{ \frac{Y_i(t)}{\hat{G}^w(x;t)} - \hat{S}^w(x;t) \right\} \middle| X_i = x \right] \\ &\quad = e_0^w(x) \left\{ \mathbb{E}\left[\frac{Y_i(t)}{\hat{G}^w(x;t)} \middle| X_i = x, W_i = w \right] - \hat{S}^w(x;t) \right\}. \end{aligned}$$

Since $Y_i(t) = \mathbb{I}(T_i > t, \mathcal{C}_i > t)$, by noninformative censoring,

$$\begin{aligned} \mathbb{E}\left[\frac{Y_i(t)}{\hat{G}^w(x;t)} \middle| X_i = x, W_i = w \right] &= \frac{P(T_i > t, \mathcal{C}_i > t \mid X_i = x, W_i = w)}{\hat{G}^w(x;t)} \\ &= \frac{P(T_i > t \mid X_i = x, W_i = w)\, P(\mathcal{C}_i > t \mid X_i = x, W_i = w)}{\hat{G}^w(x;t)} \\ &= \frac{S_0^w(x;t)\, G_0^w(x;t)}{\hat{G}^w(x;t)}. \end{aligned}$$

Hence,

$$\mathbb{E}\{\hat{\pi}_i^w(t) \mid X_i = x\} = \hat{S}^w(x;t) + \frac{e_0^w(x)}{\hat{e}^w(x)} \left\{ \frac{S_0^w(x;t)\, G_0^w(x;t)}{\hat{G}^w(x;t)} - \hat{S}^w(x;t) \right\}.$$

Subtracting $S_0^w(x;t)$ from both sides gives

$$\begin{aligned} &\mathbb{E}\{\hat{\pi}_i^w(t) \mid X_i = x\} - S_0^w(x;t) \\ &\quad = \hat{S}^w(x;t) - S_0^w(x;t) + \frac{e_0^w(x)}{\hat{e}^w(x)} \left\{ \frac{S_0^w(x;t)\, G_0^w(x;t)}{\hat{G}^w(x;t)} - \hat{S}^w(x;t) \right\}. \end{aligned}$$

Add and subtract $\frac{e_0^w(x)}{\hat{e}^w(x)} S_0^w(x;t)$ to obtain

$$
\begin{aligned}
&\mathbb{E}\{\hat{\pi}_i^w(t) \mid X_i = x\} - S_0^w(x;t) \\
&\quad = \left\{1 - \frac{e_0^w(x)}{\hat{e}^w(x)}\right\} \left\{\hat{S}^w(x;t) - S_0^w(x;t)\right\} + \frac{e_0^w(x)}{\hat{e}^w(x)} \left\{\frac{S_0^w(x;t)\, G_0^w(x;t)}{\hat{G}^w(x;t)} - S_0^w(x;t)\right\} \\
&\quad = \frac{\hat{e}^w(x) - e_0^w(x)}{\hat{e}^w(x)} \left\{\hat{S}^w(x;t) - S_0^w(x;t)\right\} + \frac{e_0^w(x)}{\hat{e}^w(x)} \frac{S_0^w(x;t)}{\hat{G}^w(x;t)} \left\{G_0^w(x;t) - \hat{G}^w(x;t)\right\}.
\end{aligned}
$$

Now use

$$
\hat{\varphi}_i(t) = \hat{\pi}_i^1(t) - \hat{\pi}_i^0(t), \qquad \varphi_i^0(t) = \pi_{i,0}^1(t) - \pi_{i,0}^0(t),
$$

together with

$$
\mathbb{E}\{\pi_{i,0}^w(t) \mid X_i = x\} = S_0^w(x;t), \qquad w = 0, 1,
$$

to obtain

$$
\begin{aligned}
\mathbb{E}\big[\hat{\varphi}_i(t) - \varphi_i^0(t) \mid X_i = x\big] = {} & \Big(\mathbb{E}\{\hat{\pi}_i^1(t) \mid X_i = x\} - S_0^1(x;t)\Big) \\
& - \Big(\mathbb{E}\{\hat{\pi}_i^0(t) \mid X_i = x\} - S_0^0(x;t)\Big),
\end{aligned}
$$

which yields (5).

It remains to bound the right-hand side uniformly in x. By positivity and uniform consistency of $\hat{e}^w$ and $\hat{G}^w$ (Assumption 6) , with probability tending to one,

$$
\inf_x \hat{e}^w(x) \geq c/2, \qquad \inf_x \hat{G}^w(x;t) \geq c/2.
$$

Also $0 \leq S_0^w(x;t) \leq 1$. Therefore,

$$
\begin{aligned}
\sup_x \left| \frac{\hat{e}^w(x) - e_0^w(x)}{\hat{e}^w(x)} \left\{\hat{S}^w(x;t) - S_0^w(x;t)\right\} \right| &= O_p\Big(r_n^{(e)} r_n^{(S)}\Big), \\
\sup_x \left| \frac{e_0^w(x)}{\hat{e}^w(x)} \frac{S_0^w(x;t)}{\hat{G}^w(x;t)} \left\{G_0^w(x;t) - \hat{G}^w(x;t)\right\} \right| &= O_p\Big(r_n^{(G)}\Big).
\end{aligned}
$$

Summing over $w = 0, 1$ yields

$$
\sup_x \big|\mathbb{E}\big[\hat{\varphi}_i(t) - \varphi_i^0(t) \mid X_i = x\big]\big| = O_p\Big(r_n^{(e)} r_n^{(S)} + r_n^{(G)}\Big).
$$

If $\hat{G}^w = G_0^w$, the second term vanishes and the bound reduces to

$$
O_p\Big(r_n^{(e)} r_n^{(S)}\Big).
$$

This completes the proof.

Proof of Theorem 4: For notational simplicity, write

$$\hat{\varphi}_i = \hat{\varphi}_i(t), \qquad \varphi_i^0 = \varphi_i^0(t), \qquad \tau_0(x) = \tau_0(x;t).$$

Define the conditional mean of the estimated pseudo-outcome by

$$m_n(x) := \mathbb{E}[\hat{\varphi}_i \mid X_i = x].$$

Then

$$\hat{\tau}(x) - \tau_0(x) = \{\hat{\tau}(x) - m_n(x)\} + \{m_n(x) - \tau_0(x)\},$$

and therefore

$$\|\hat{\tau} - \tau_0\|_{L_2(P_X)} \le \|\hat{\tau} - m_n\|_{L_2(P_X)} + \|m_n - \tau_0\|_{L_2(P_X)}. \tag{6}$$

We study the two terms separately.

By definition of τ_0,

$$\tau_0(x) = \mathbb{E}[\varphi_i^0 \mid X_i = x].$$

Hence

$$m_n(x) - \tau_0(x) = \mathbb{E}[\hat{\varphi}_i - \varphi_i^0 \mid X_i = x].$$

By Theorem 3,

$$\sup_x |m_n(x) - \tau_0(x)| = \sup_x \big|\mathbb{E}[\hat{\varphi}_i - \varphi_i^0 \mid X_i = x]\big| = O_p\big(r_n^{(e)} r_n^{(S)} + r_n^{(G)}\big).$$

Since $r_n^{(e)} r_n^{(S)} + r_n^{(G)} \to 0$,

$$\sup_x |m_n(x) - \tau_0(x)| = o_p(1).$$

Therefore,

$$\|m_n - \tau_0\|_{L_2(P_X)} \le \sup_x |m_n(x) - \tau_0(x)| = o_p(1). \tag{7}$$

For any measurable function f,

$$R_n(f) := \mathbb{E}\big[(\hat{\varphi}_i - f(X_i))^2\big] < \infty$$

by Assumption 7

Expanding the square conditional on X_i,

$$\begin{aligned} R_n(f) &= \mathbb{E}\Big(\mathbb{E}\big[(\hat{\varphi}_i - f(X_i))^2 \mid X_i\big]\Big) \\ &= \mathbb{E}\Big(\mathrm{Var}(\hat{\varphi}_i \mid X_i) + \{m_n(X_i) - f(X_i)\}^2\Big). \end{aligned}$$

Thus,

$$R_n(f) = \mathbb{E}[\mathrm{Var}(\hat{\varphi}_i \mid X_i)] + \|f - m_n\|_{L_2(P_X)}^2.$$

Therefore m_n is the unique population minimizer of $R_n(f)$ over all square-integrable functions, and over $\mathcal{F}_n$ the excess risk satisfies

$$R_n(f) - R_n(m_n) = \|f - m_n\|_{L_2(P_X)}^2. \tag{8}$$

Let

$$f_n^* \in \arg\min_{f \in \mathcal{F}_n} R_n(f)$$

be a population risk minimizer over $\mathcal{F}_n$. Since $\hat{\tau}$ minimizes the empirical risk,

$$\mathbb{P}_n[(\hat{\varphi}_i - \hat{\tau}(X_i))^2] \leq \mathbb{P}_n[(\hat{\varphi}_i - f_n^*(X_i))^2].$$

Subtract $R_n(\hat{\tau})$ from the left-hand side and $R_n(f_n^*)$ from the right-hand side:

$$R_n(\hat{\tau}) - R_n(f_n^*) \leq \Big\{ R_n(\hat{\tau}) - \mathbb{P}_n[(\hat{\varphi}_i - \hat{\tau}(X_i))^2] \Big\} + \Big\{ \mathbb{P}_n[(\hat{\varphi}_i - f_n^*(X_i))^2] - R_n(f_n^*) \Big\}.$$

Hence

$$R_n(\hat{\tau}) - R_n(f_n^*) \leq 2 \sup_{f \in \mathcal{F}_n} \big| \mathbb{P}_n[(\hat{\varphi}_i - f(X_i))^2] - R_n(f) \big| = o_p(1)$$

by Assumption 9. Using (8),

$$\|\hat{\tau} - m_n\|_{L_2(P_X)}^2 \leq \|f_n^* - m_n\|_{L_2(P_X)}^2 + o_p(1). \tag{9}$$

We now relate m_n to τ_0. For any $f \in \mathcal{F}_n$,

$$\|f - m_n\|_{L_2(P_X)} \leq \|f - \tau_0\|_{L_2(P_X)} + \|\tau_0 - m_n\|_{L_2(P_X)}.$$

Taking infima over $f \in \mathcal{F}_n$ and using Assumption 8 together with (7), we obtain

$$\inf_{f \in \mathcal{F}_n} \|f - m_n\|_{L_2(P_X)} \leq \inf_{f \in \mathcal{F}_n} \|f - \tau_0\|_{L_2(P_X)} + \|\tau_0 - m_n\|_{L_2(P_X)} =$$

$$\inf_{f \in \mathcal{F}_n} \|f - \tau_0\|_{L_\infty(P_X)} + o_p(1) = o_p(1).$$

Since f_n^* minimizes $R_n(f)$ over $\mathcal{F}_n$, equivalently it minimizes $\|f - m_n\|_{L_2(P_X)}^2$ over $\mathcal{F}_n$. Therefore,

$$\|f_n^* - m_n\|_{L_2(P_X)} = o_p(1). \tag{10}$$

Combining (9) and (10) yields

$$\|\hat{\tau} - m_n\|_{L_2(P_X)} = o_p(1).$$

Finally, substitute this and (7) into (6):

$$\|\hat{\tau} - \tau_0\|_{L_2(P_X)} \leq \|\hat{\tau} - m_n\|_{L_2(P_X)} + \|m_n - \tau_0\|_{L_2(P_X)} = o_p(1).$$

Hence

$$\|\hat{\tau}(\cdot; t) - \tau_0(\cdot; t)\|_{L_2(P_X)} \xrightarrow{p} 0.$$

This completes the proof.

Proof of Theorem 5: Write

$$\hat{\varphi}_i = \hat{\varphi}_i(t), \qquad \varphi_i^0 = \varphi_i^0(t), \qquad \tau_0(x) = \tau_0(x;t),$$

and define

$$m_n(x) := \mathbb{E}(\hat{\varphi}_i \mid X_i = x).$$

Because $\hat{\tau}(\cdot;t)$ is obtained by regressing the cross-fitted pseudo-outcome on X_i, the relevant population regression target is m_n, not τ_0 directly. We therefore decompose

$$\|\hat{\tau} - \tau_0\|_{L_2(P_X)} \le \|\hat{\tau} - m_n\|_{L_2(P_X)} + \|m_n - \tau_0\|_{L_2(P_X)}. \tag{11}$$

We first control the second term using the explicit construction of the pseudo-outcome. By definition,

$$m_n(x) - \tau_0(x) = \mathbb{E}\{\hat{\varphi}_i - \varphi_i^0 \mid X_i = x\},$$

since

$$\tau_0(x) = \mathbb{E}\{\varphi_i^0 \mid X_i = x\}.$$

By Theorem 3,

$$\sup_x |m_n(x) - \tau_0(x)| = O_p\big(r_n^{(e)} r_n^{(S)} + r_n^{(G)}\big).$$

Hence

$$\|m_n - \tau_0\|_{L_2(P_X)} \le \sup_x |m_n - \tau_0| = O_p\big(r_n^{(e)} r_n^{(S)} + r_n^{(G)}\big), \tag{12}$$

which accommodates censoring estimation error with $r_n^{(G)}$.

Next, consider the population risk associated with the constructed pseudo-outcome,

$$R_n(f) := \mathbb{E}\big[(\hat{\varphi}_i - f(X_i))^2\big].$$

Since $m_n(x) = \mathbb{E}(\hat{\varphi}_i \mid X_i = x)$, conditioning on X_i gives

$$R_n(f) = R_n(m_n) + \|f - m_n\|_{L_2(P_X)}^2.$$

Thus m_n is the population least-squares target for the regression step, and

$$R_n(f) - R_n(m_n) = \|f - m_n\|_{L_2(P_X)}^2. \tag{13}$$

We now use the fact that τ_0 is Hölder smooth. By Assumption 8, there exists $f_n^\star \in \mathcal{F}_n$ such that

$$\|f_n^\star - \tau_0\|_{L_\infty(P_X)} \le C n^{-\beta/(2\beta+d)}.$$

Therefore,

$$\|f_n^\star - m_n\|_{L_2(P_X)} \le \|f_n^\star - \tau_0\|_{L_2(P_X)} + \|\tau_0 - m_n\|_{L_2(P_X)} = O_p\Big(n^{-\beta/(2\beta+d)} + r_n^{(e)} r_n^{(S)} + r_n^{(G)}\Big),$$

where we used (12) and the density bound in Assumption 3. Consequently,

$$R_n(f_n^\star)-R_n(m_n) = \|f_n^\star-m_n\|_{L_2(P_X)}^2 = O_p\Big(n^{-2\beta/(2\beta+d)} + \{r_n^{(e)}r_n^{(S)} + r_n^{(G)}\}^2\Big). \tag{14}$$

Because $\hat\tau$ is an approximate empirical minimizer, it follows that

$$\mathbb{P}_n\big[(\hat\varphi_i - \hat\tau(X_i))^2\big] \le \mathbb{P}_n\big[(\hat\varphi_i - f_n^\star(X_i))^2\big] + \delta_n^2,$$

by Assumption 10. Adding and subtracting population risks yields

$$\begin{aligned}
R_n(\hat\tau) - R_n(f_n^\star) &\le \big\{R_n(\hat\tau) - \mathbb{P}_n[(\hat\varphi_i - \hat\tau(X_i))^2]\big\} \\
&\quad + \big\{\mathbb{P}_n[(\hat\varphi_i - f_n^\star(X_i))^2] - R_n(f_n^\star)\big\} + \delta_n^2 \\
&\le 2\sup_{f\in\mathcal{F}_n}\big|\mathbb{P}_n\big[(\hat\varphi_i - f(X_i))^2\big] - R_n(f)\big| + \delta_n^2 \\
&= O_p(\rho_n) + \delta_n^2,
\end{aligned}$$

where the last equality is due to Assumption 9. Combining this with (14), we obtain

$$R_n(\hat\tau) - R_n(m_n) = O_p\Big(n^{-2\beta/(2\beta+d)} + \rho_n + \delta_n^2 + \{r_n^{(e)}r_n^{(S)} + r_n^{(G)}\}^2\Big).$$

Applying (13),

$$\|\hat\tau - m_n\|_{L_2(P_X)}^2 = O_p\Big(n^{-2\beta/(2\beta+d)} + \rho_n + \delta_n^2 + \{r_n^{(e)}r_n^{(S)} + r_n^{(G)}\}^2\Big),$$

and hence

$$\|\hat\tau - m_n\|_{L_2(P_X)} = O_p\Big(n^{-\beta/(2\beta+d)} + \rho_n^{1/2} + \delta_n + r_n^{(e)}r_n^{(S)} + r_n^{(G)}\Big). \tag{15}$$

Finally, combine (11), (12), and (15) to conclude

$$\|\hat\tau - \tau_0\|_{L_2(P_X)} = O_p\Big(n^{-\beta/(2\beta+d)} + \rho_n^{1/2} + \delta_n + r_n^{(e)}r_n^{(S)} + r_n^{(G)}\Big).$$

This completes the proof.

Proof of Lemma 1 We give a constructive proof based on a local polynomial approximation in the time direction. We aim to show that the coefficient functions in this expansion inherit the β_x-Hölder smoothness of $\tau_0(x,t)$ in the covariate direction.

Write

$$\beta_t = q_t + \alpha_t, \qquad q_t = \lfloor \beta_t \rfloor, \qquad \alpha_t \in (0,1].$$

Fix an integer $K \ge 1$. Partition $[0,1]$ into K equal subintervals

$$I_r = \Big[\frac{r-1}{K}, \frac{r}{K}\Big), \qquad r = 1,\dots,K-1, \qquad I_K = \Big[\frac{K-1}{K}, 1\Big].$$

Let u_r denote the midpoint of I_r. For each interval I_r, we approximate $\tau_0(x,t)$ in the time variable t by its Taylor polynomial of order q_t around u_r.

For $r = 1, \ldots, K$ and $\ell = 0, \ldots, q_t$, define

$$B_{r,\ell}(t) := (t - u_r)^\ell \mathbf{1}\{t \in I_r\},$$

and define the corresponding coefficient functions

$$b_{r,\ell}(x) := \frac{\partial_t^\ell \tau_0(x, u_r)}{\ell!}, \qquad x \in \mathcal{X}.$$

Then, for $t \in I_r$,

$$\sum_{\ell=0}^{q_t} b_{r,\ell}(x) B_{r,\ell}(t) = \sum_{\ell=0}^{q_t} \frac{\partial_t^\ell \tau_0(x, u_r)}{\ell!} (t - u_r)^\ell,$$

which is the Taylor polynomial of $\tau_0(x, \cdot)$ at u_r.

We now verify that, uniformly over r and ℓ, the function $b_{r,\ell}$ belongs to a β_x-Hölder ball on $\mathcal{X}$.

Write

$$\beta_x = q_x + \alpha_x, \qquad q_x = \lfloor \beta_x \rfloor, \qquad \alpha_x \in (0, 1].$$

Fix $r \in \{1, \ldots, K\}$ and $\ell \in \{0, \ldots, q_t\}$. By definition,

$$b_{r,\ell}(x) = \frac{\partial_t^\ell \tau_0(x, u_r)}{\ell!}.$$

For any multi-index ν with $|\nu| \le q_x$,

$$D_x^\nu b_{r,\ell}(x) = \frac{D_x^\nu \partial_t^\ell \tau_0(x, u_r)}{\ell!}.$$

Because $\tau_0 \in \mathcal{H}^{\beta_x, \beta_t}(M)$, Definition 2 implies that all such derivatives exist and are uniformly bounded:

$$\sup_{x \in \mathcal{X}} |D_x^\nu b_{r,\ell}(x)| \le \frac{1}{\ell!} \sup_{x \in \mathcal{X}} |D_x^\nu \partial_t^\ell \tau_0(x, u_r)| \le \frac{M}{\ell!} \le M.$$

Next, for any multi-index ν with $|\nu| = q_x$, the Hölder condition in the x-direction yields

$$\frac{|D_x^\nu b_{r,\ell}(x) - D_x^\nu b_{r,\ell}(y)|}{\|x - y\|^{\alpha_x}} = \frac{|D_x^\nu \partial_t^\ell \tau_0(x, u_r) - D_x^\nu \partial_t^\ell \tau_0(y, u_r)|}{\ell! \, \|x - y\|^{\alpha_x}} \le \frac{M}{\ell!} \le M.$$

Thus each $b_{r,\ell}$ lies in a β_x-Hölder class on $\mathcal{X}$, with Hölder radius bounded by a constant depending only on M, β_x, β_t. In particular, there exists a constant $C_M > 0$, depending only on M, β_x, β_t, such that

$$b_{r,\ell} \in \mathcal{H}^{\beta_x}(C_M) \qquad \text{for all } r = 1, \ldots, K, \ \ell = 0, \ldots, q_t.$$

Fix $j \in \{1, \ldots, J\}$, and let $r(j)$ denote the unique index such that $t_j \in I_{r(j)}$. Define the local polynomial approximation at the grid point t_j by

$$P_j(x) := \sum_{\ell=0}^{q_t} b_{r(j),\ell}(x) B_{r(j),\ell}(t_j) = \sum_{\ell=0}^{q_t} \frac{\partial_t^\ell \tau_0(x, u_{r(j)})}{\ell!} (t_j - u_{r(j)})^\ell.$$

We claim that

$$\sup_{x\in\mathcal{X}} |\tau_0(x,t_j) - P_j(x)| \le CK^{-\beta_t},$$

uniformly in j.

To see this, fix $x \in \mathcal{X}$. By Taylor's theorem in the one-dimensional variable t, applied to the function $t \mapsto \tau_0(x,t)$ around the point $u_{r(j)}$, there exists a point $\xi_{x,j}$ between $u_{r(j)}$ and t_j such that

$$\tau_0(x,t_j) - P_j(x) = \frac{\partial_t^{q_t}\tau_0(x,\xi_{x,j}) - \partial_t^{q_t}\tau_0(x,u_{r(j)})}{q_t!}(t_j - u_{r(j)})^{q_t}.$$

Because $\tau_0 \in \mathcal{H}^{\beta_x,\beta_t}(M)$, the derivative $\partial_t^{q_t}\tau_0(x,\cdot)$ is α_t-Hölder uniformly in x, so

$$|\partial_t^{q_t}\tau_0(x,\xi_{x,j}) - \partial_t^{q_t}\tau_0(x,u_{r(j)})| \le M|\xi_{x,j} - u_{r(j)}|^{\alpha_t} \le M|t_j - u_{r(j)}|^{\alpha_t}.$$

Therefore,

$$|\tau_0(x,t_j) - P_j(x)| \le C|t_j - u_{r(j)}|^{q_t+\alpha_t} = C|t_j - u_{r(j)}|^{\beta_t}.$$

Since both t_j and $u_{m(j)}$ lie in the same interval $I_{m(j)}$, whose length is K^{-1}, we have

$$|t_j - u_{m(j)}| \le K^{-1}.$$

Hence

$$\sup_{x\in\mathcal{X}} |\tau_0(x,t_j) - P_j(x)| \le CK^{-\beta_t},$$

uniformly in j. Since P_X is a probability measure,

$$\|\tau_0(\cdot,t_j) - P_j\|^2_{L_2(P_X)} \le \sup_{x\in\mathcal{X}} |\tau_0(x,t_j) - P_j(x)|^2 \le CK^{-2\beta_t}.$$

Taking the supremum over j gives

$$\sup_{1\le j\le J} \|\tau_0(\cdot,t_j) - P_j\|^2_{L_2(P_X)} \le CK^{-2\beta_t}.$$

So far we have constructed $(q_t+1)K$ basis functions $B_{r,\ell}$ and the same number of coefficient functions $b_{r,\ell}$. Each approximant P_j can be written as

$$P_j(x) = \sum_{r=1}^{K}\sum_{\ell=0}^{q_t} b_{r,\ell}(x)B_{r,\ell}(t_j).$$

Relabel the collection

$$\{B_{m,\ell} : 1 \le r \le K,\ 0 \le \ell \le q_t\}$$

as

$$\psi_1, \ldots, \psi_{(q_t+1)K},$$

and similarly relabel the coefficient functions

$$\{b_{r,\ell} : 1 \le m \le K,\ 0 \le \ell \le q_t\}$$

as

$$b_1, \ldots, b_{(q_t+1)K}.$$

Now define

$$m := (q_t + 1)K.$$

Since q_t depends only on β_t, this is only a constant rescaling of K, and thus

$$K^{-2\beta_t} \asymp m^{-2\beta_t}.$$

Absorbing constants into C, we obtain

$$\sup_{1 \le j \le J} \left\| \tau_0(\cdot; t_j) - \sum_{k=1}^{m} b_k(\cdot) B_k(t_j) \right\|_{L_2(P_X)}^2 \le C m^{-2\beta_t}.$$

Finally,

$$\frac{1}{J} \sum_{j=1}^{J} \left\| \tau_0(\cdot; t_j) - \sum_{k=1}^{m} b_k(\cdot) B_k(t_j) \right\|_{L_2(P_X)}^2 \le C m^{-2\beta_t}.$$

This completes the proof.

Proof of Theorem 6: For $j = 1, \ldots, J$, let

$$\bar{\varphi}_j(x) = \mathbb{E}\{\hat{\varphi}_i(t_j) \mid X_i = x\}$$

By Theorem 3

$$\sup_x |\bar{\varphi}_j(x) - \tau_0(x; t_j)| = O_p\left(r_n^{(e)} r_n^{(S)} + r_n^{(G)} \right)$$

Therefore

$$\frac{1}{J} \sum_{j=1}^{J} \|\bar{\varphi}_j(\cdot) - \tau_0(\cdot; t_j)\|_{L_2(P_X)}^2 = O_p\left(\left\{ r_n^{(e)} r_n^{(S)} + r_n^{(G)} \right\}^2 \right) \qquad (16)$$

For $f \in \mathcal{F}_{n,m}$, let

$$\tau_f(x; t) = \sum_{k=1}^{m} f_k(x) B_k(t)$$

We then define the empirical and population risks as

$$R_{n,J}(f) = \mathbb{P}_n \left[\frac{1}{J} \sum_{j=1}^{J} \{\hat{\varphi}_i(t_j) - \tau_f(X_i; t_j)\}^2 \right]$$

and

$$R_J(f) = \mathbb{E} \left[\frac{1}{J} \sum_{j=1}^{J} \{\hat{\varphi}_i(t_j) - \tau_f(X_i; t_j)\}^2 \right]$$

We can write

$$\begin{aligned}&\mathbb{E}\left[\{\hat{\varphi}_i(t_j)-\tau_f(X_i;t_j)\}^2\right]\\&=\mathbb{E}\left[\{\hat{\varphi}_i(t_j)-\bar{\varphi}_j(X_i)\}^2\right]+\|\tau_f(\cdot;t_j)-\bar{\varphi}_j(X_i)\|^2_{L_2(P_X)}.\end{aligned}$$

where the interaction term is zero because $\bar{\varphi}_j(X_i)$ is the expectation of $\hat{\varphi}_i(t_j)$ conditional on X_i. Then,

$$R_J(f)=C_{n,J}+\frac{1}{J}\sum_{j=1}^{J}\|\tau_f(\cdot;t_j)-\bar{\varphi}_j(\cdot)\|^2_{L_2(P_X)}, \tag{17}$$

where

$$C_{n,J}=\frac{1}{J}\sum_{j=1}^{J}\mathbb{E}\left[\{\hat{\varphi}_i(t_j)-\bar{\varphi}_j(X_i)\}^2\right]$$

does not depend on f.

By Lemma 1, for fixed m, there exist basis functions $B_1(t),\ldots,B_m(t)$ and coefficient functions $b_1,\ldots,b_m\in\mathcal{H}^{\beta_x}(C)$ such that

$$\sum_{j=1}^{J}\left\|\tau_0(\cdot;t_j)-\sum_{k=1}^{m}b_k(\cdot)B_m(t_j)\right\|^2_{L_2(P_X)}\lesssim m^{-2\beta_t}.$$

Since each b_k is β_x-Hölder, by Assumption 8, there exists $f^\star=(f_1^\star,\ldots,f_m^\star)^\top\in\mathcal{F}_{n,m}$ such that

$$\max_{k=1,\ldots,m}\|f_k^\star-b_k\|^2_{L_2(P_X)}\lesssim n^{-2\beta_x/(2\beta_x+d)}.$$

We define the optimization error as

$$\delta^2_{n,J,m}=R_{n,J}(\hat{f})-\inf_{f\in\mathcal{F}_{n,m}}R_{n,J}(f)$$

then for $f^\star$, we have

$$\begin{aligned}R_J(\hat{f})-R_J(f^\star)&\le\left\{R_J(\hat{f})-R_{n,J}(\hat{f})\right\}\\&\quad+\{R_{n,J}(f^\star)-R_J(f^\star)\}+\delta^2_{n,J,m}\\&\le 2\sup_{f\in\mathcal{F}_{n,m}}|R_{n,J}(f)-R_J(f)|+\delta^2_{n,J,m}.\end{aligned}$$

Using (17), we have

$$\begin{aligned}&\frac{1}{J}\sum_{j=1}^{J}\left\|\tau_{\hat{f}}(\cdot;t_j)-\bar{\varphi}_j(\cdot)\right\|^2_{L_2(P_X)}\\&\quad\le\frac{1}{J}\sum_{j=1}^{J}\|\tau_{f^\star}(\cdot;t_j)-\bar{\varphi}_j(\cdot)\|^2_{L_2(P_X)}+2\sup_{f\in\mathcal{F}_{n,m}}|R_{n,J}(f)-R_J(f)|+\delta^2_{n,J,m}.\end{aligned} \tag{18}$$

We first bound $\sum_{j=1}^{J} \left\| \tau_{\hat{f}}(\cdot;t_j) - \bar{\varphi}_j(\cdot) \right\|_{L_2(P_X)}^2$. For the basis functions $B_1(t), \ldots, B_m(t)$ and coefficient functions $b_1, \ldots, b_m$ satisfy the conditions in Lemma 1, let

$$\tau_b(x;t) = \sum_{k=1}^{m} b_k(x) B_k(t),$$

we have

$$\tau_{f^\star}(x;t_j) - \tau_b(x;t_j) = \sum_{k=1}^{m} \left\{ f_k^\star(x) - b_k(x) \right\} B_k(t_j).$$

By the spline stability condition,

$$\lambda_{\max}\left(\frac{1}{J} \mathbf{B}^\top \mathbf{B} \right) \le \frac{C_B}{m},$$

and therefore

$$\begin{aligned} &\frac{1}{J} \sum_{j=1}^{J} \| \tau_{f^\star}(\cdot;t_j) - \tau_b(\cdot;t_j) \|_{L_2(P_X)}^2 \\ &\quad \le \frac{C_B}{m} \sum_{k=1}^{m} \| f_k^\star - b_k \|_{L_2(P_X)}^2 \\ &\quad \lesssim n^{-2\beta_x/(2\beta_x+d)}. \end{aligned} \tag{19}$$

Combine this with

$$\sum_{j=1}^{J} \| \tau_0(\cdot;t_j) - \tau_b(\cdot;t_j) \|_{L_2(P_X)}^2 \lesssim m^{-2\beta_t}.$$

we have

$$\frac{1}{J} \sum_{j=1}^{J} \| \tau_{f^\star}(\cdot;t_j) - \tau_0(\cdot;t_j) \|_{L_2(P_X)}^2 \lesssim m^{-2\beta_t} + n^{-2\beta_x/(2\beta_x+d)}. \tag{20}$$

Next, by

$$\tau_{f^\star}(\cdot;t_j) - \bar{\varphi}_j(\cdot) = \left\{ \tau_{f^\star}(\cdot;t_j) - \tau_0(\cdot;t_j) \right\} + \left\{ \tau_0(\cdot;t_j) - \bar{\varphi}_j(\cdot) \right\},$$

together with (16) and (20),

$$\begin{aligned} &\frac{1}{J} \sum_{j=1}^{J} \| \tau_{f^\star}(\cdot;t_j) - \bar{\varphi}_j(\cdot) \|_{L_2(P_X)}^2 \\ &\quad = O_p\left(m^{-2\beta_t} + n^{-2\beta_x/(2\beta_x+d)} + \left\{ r_n^{(e)} r_n^{(S)} + r_n^{(G)} \right\}^2 \right). \end{aligned} \tag{21}$$

We then bound the empirical process fluctuation $\sup_{f \in \mathcal{F}_{n,m}} |R_{n,J}(f) - R_J(f)|$. For the scalar output DNN $\mathcal{F}_{n,1}$, let ρ_n denote the corresponding empirical

process fluctuation from Theorem 5. For $\tau, \tilde{\tau} \in \mathcal{T}_{n,m}$, define the aggregated metric

$$d_J^2(\tau, \tilde{\tau}) = \frac{1}{J}\sum_{j=1}^{J} \|\tau(\cdot; t_j) - \tilde{\tau}(\cdot; t_j)\|_{L_2(P_X)}^2.$$

Since $\mathcal{F}_{n,m} \subseteq \mathcal{F}_{n,1}^m$ and, by the basis stability condition,

$$d_J^2(\tau_f, \tau_{\tilde{f}}) \le \frac{C_B}{m}\sum_{k=1}^{m} \|f_k - \tilde{f}_k\|_{L_2(P_X)}^2,$$

the metric entropy of the induced joint class satisfies

$$\log N(\varepsilon, \mathcal{T}_{n,m}, d_J) \le m \log N(c\varepsilon, \mathcal{F}_{n,1}, L_2(P_X))$$

for some constant $c > 0$. Applying the standard symmetrization, contraction, and entropy-integral argument for the squared-loss class, together with the entropy condition on $\mathcal{F}_{n,1}$, gives

$$\sup_{f \in \mathcal{F}_{n,m}} |R_{n,J}(f) - R_J(f)| = O_p\left(\sqrt{m}\,\rho_n\right).$$

Combining this together with (21) and (18), we have

$$\begin{aligned} &\frac{1}{J}\sum_{j=1}^{J} \left\|\tau_{\hat{f}}(\cdot; t_j) - \bar{\varphi}_j(\cdot)\right\|_{L_2(P_X)}^2 \\ &\quad = O_p\left(m^{-2\beta_t} + n^{-2\beta_x/(2\beta_x+d)} + \sqrt{m}\,\rho_n + \delta_{n,J,m}^2 + \left\{r_n^{(e)} r_n^{(S)} + r_n^{(G)}\right\}^2\right). \end{aligned} \tag{22}$$

Finally, since

$$\hat{\tau}(\cdot; t_j) - \tau_0(\cdot; t_j) = \{\hat{\tau}(\cdot; t_j)\bar{\varphi}_j(\cdot)\} + \{\bar{\varphi}_j(\cdot) - \tau_0(\cdot; t_j)\},$$

with (16) and (22), we obtain

$$\begin{aligned} &\frac{1}{J}\sum_{j=1}^{J} \|\hat{\tau}(\cdot; t_j) - \tau_0(\cdot; t_j)\|_{L_2(P_X)}^2 \\ &\quad = O_p\left(m^{-2\beta_t} + n^{-2\beta_x/(2\beta_x+d)} + \sqrt{m}\,\rho_n + \delta_{n,J,m}^2 + \left\{r_n^{(e)} r_n^{(S)} + r_n^{(G)}\right\}^2\right). \end{aligned}$$

This completes the proof.

Additional Simulation Results

To further investigate the finite-sample behavior observed in Case 1, we conducted an additional set of simulations under a simplified setting with lower-dimensional covariates ($d = 10$) and a larger training sample size ($N = 2000$). This setting is closer to those often considered in prior methodological work and therefore provides a useful benchmark for assessing whether the

performance patterns in Case 1 were driven by limited sample size and model complexity. The results are reported in Table A.1.

As shown in Table A.1, all methods worked better under this easier setting, and several competing approaches achieve noticeably smaller MSE than DSL. In particular, PSDRL-NFT-BART attains the lowest MSE overall, approximately 0.014 across all choices of J, while X-Learner and causal survival forest also perform strongly, with MSEs around 0.020 and 0.021, respectively. DRL-NFT-BART improves substantially as the number of evaluation time points increases, with MSE decreasing from 0.042 at $J = 1$ to 0.022 at $J = 100$. By comparison, DSL yields MSEs of 0.042, 0.046, and 0.047 for $J = 1, 50, 100$, respectively, which are somewhat larger but remain reasonably stable across grid sizes.

A notable feature of DSL is its very small bias. Its bias remains close to zero across all three values of J, around 0.005, and is clearly smaller than that of several competitors, including M-Learner, PSDRL-NFT-BART, and causal survival forest, all of which exhibit substantially larger systematic bias. R-Learner and DRL-NFT-BART also have relatively small bias, but their MSE is not uniformly better than that of the strongest competitors. Taken together, these results suggest that in a simpler and more data-rich setting, competing methods can achieve very strong predictive accuracy, sometimes surpassing DSL in MSE, while DSL continues to provide stable estimation with consistently low bias. This supports our interpretation that the larger performance gaps seen in Case 1 may be primarily attributable to the more challenging finite-sample and high-complexity setting.

Table A.1 Performance comparison under simplified setting

	$J = 1$	$J = 50$	$J = 100$
**MSE**			
Deep Survival Learner	0.042(0.041, 0.043)	0.046(0.045, 0.048)	0.047(0.045, 0.048)
M-Learner	0.049(0.047, 0.051)	0.049(0.047, 0.051)	0.049(0.047, 0.051)
R-Learner	0.049(0.047, 0.051)	0.051(0.049, 0.053)	0.048(0.046, 0.050)
X-Learner	0.020(0.019, 0.021)	0.020(0.019, 0.021)	0.020(0.019, 0.021)
DRL-NFT-BART	0.042(0.026, 0.057)	0.025(0.020, 0.030)	0.022(0.020, 0.024)
PSDRL-NFT-BART	0.014(0.013, 0.015)	0.014(0.014, 0.015)	0.014(0.014, 0.015)
Causal Survival Forest	0.021(0.020, 0.022)	0.021(0.020, 0.022)	0.021(0.020, 0.022)
**Bias**			
Deep Survival Learner	0.005(0.001, 0.010)	0.005(0.000, 0.009)	0.005(0.000, 0.009)
M-Learner	0.109(0.105, 0.113)	0.109(0.105, 0.113)	0.109(0.105, 0.113)
R-Learner	0.012(0.006, 0.017)	0.007(0.000, 0.014)	0.012(0.006, 0.018)
X-Learner	0.037(0.031, 0.043)	0.041(0.036, 0.046)	0.038(0.032, 0.043)
DRL-NFT-BART	0.013(0.003, 0.023)	0.012(0.005, 0.020)	0.013(0.007, 0.020)
PSDRL-NFT-BART	0.083(0.079, 0.087)	0.084(0.081, 0.088)	0.084(0.080, 0.088)
Causal Survival Forest	0.107(0.103, 0.111)	0.107(0.102, 0.111)	0.107(0.102, 0.111)

[1] The simulation setting uses a training sample size of $N = 2000$ and a testing sample size of 400, with covariate dimension $d = 10$ and approximately 35% censoring.

[2] Mean squared error and 95% Monte Carlo confidence intervals are computed based on 200 simulation replicates.

[3] Conditional average treatment effects are evaluated at $J \in \{1, 50, 100\}$ time points, equally spaced between the 20th and 80th percentiles of the observed survival time distribution.
